# Synthesis of 9-arylalkynyl- and 9-aryl-substituted benzo[*b*]quinolizinium derivatives by Palladium-mediated cross-coupling reactions

**DOI:** 10.3762/bjoc.14.161

**Published:** 2018-07-23

**Authors:** Siva Sankar Murthy Bandaru, Darinka Dzubiel, Heiko Ihmels, Mohebodin Karbasiyoun, Mohamed M A Mahmoud, Carola Schulzke

**Affiliations:** 1 Department of Chemistry and Biology, University of Siegen, Siegen, Germany; 2 Institute of Biochemistry, University of Greifswald, Greifswald, Germany

**Keywords:** DNA ligands, fluorescence, heterocycles, Pd-mediated couling reactions, quinolizinium

## Abstract

9-Arylbenzo[*b*]quinolizinium derivatives were prepared with base-free Suzuki–Miyaura coupling reactions between benzo[*b*]quinolizinium-9-trifluoroborate and selected benzenediazonium salts. In addition, the Sonogashira coupling reaction between 9-iodobenzo[*b*]quinolizinium and the arylalkyne derivatives yielded four novel 9-(arylethynyl)benzo[*b*]quinolizinium derivatives under relatively mild reaction conditions. The 9-(*N*,*N*-dimethylaminophenylethynyl)benzo[*b*]quinolizinium is only very weakly emitting, but the emission intensity increases by a factor >200 upon protonation, so that this derivative may operate as pH-sensitive light-up probe. Photometric and fluorimetric titrations of duplex and quadruplex DNA to 9-(arylethynyl)benzo[*b*]quinolizinium derivatives revealed a significant binding affinity of these compounds towards both DNA forms with binding constants of *K*_b_ = 0.2–2.2 × 10^5^ M^−1^.

## Introduction

Polycyclic cationic hetarenes are a paradigm of DNA-binding ligands whose association with the nucleic acid may affect the biological activities of the DNA [1–4]. For example, a DNA-bound heterocyclic ligand may interfere with DNA–enzyme recognition events, which are essential for DNA-based cellular processes, e.g., gene replication or transcription [1]. To this end, it was shown that DNA-binding ligands may operate as chemotherapeutic anticancer, antiviral or antibacterial drugs, for example as topoisomerase inhibitors [5]. More recently, much interest in this research area is focused on the non-canonical quadruplex DNA (G4-DNA) [6–8]. Mostly based on the principles and requirements of ligands that bind to duplex DNA, numerous G4-DNA ligands have been developed to study their selectivity and binding properties towards G4-DNA because of the biological importance of G4-DNA [9–13]. Along these lines, we and others have established the class of annelated quinolizinium derivatives as versatile ligands that bind to duplex, triplex and quadruplex DNA depending on their shape and size [14–18] and whose interaction with the nucleic acid may be used for fluorimetric detection of the latter [19–20].

To further exploit the DNA-binding properties of this specific class of cationic hetarenes, synthetic routes to novel derivatives with the desired substitution pattern and functionalization are necessary. In this context, Palladium-mediated cross-coupling reactions provide a powerful tool [21–27]; specifically, as these C–C coupling reactions have been demonstrated to be very useful for the introduction of various substituents to quinolizinium [28–33], benzo[*b*]quinolizinium [34–35] and naphthoquinolizinium [36] derivatives.

**Figure 1:**
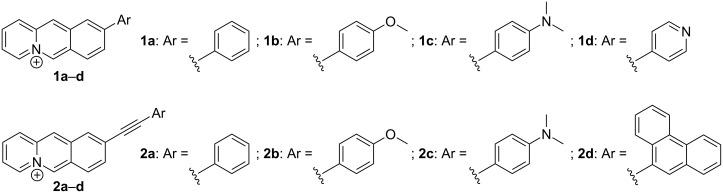
Structures of 9-substituted benzo[*b*]quinolizinium derivatives **1** and **2**.

Unfortunately, in the case of benzo[*b*]quinolizinium substrates, the presence of strong nucleophiles, and for that matter bases in general, often interferes with the Pd-mediated reaction because of the competing addition of the nucleophile at the 6-position of the substrate and subsequent ring-opening reaction [37–38]. Considering this impediment and the additional difficulties that may occur during purification of these cationic hetarenes the reaction and work-up conditions of Pd-mediated coupling reactions of benzo[*b*]quinolizinium derivatives have to be optimized [34–35]. Accordingly, we extended our studies to improve the conditions of the Suzuki–Miyaura coupling towards biaryl-type benzo[*b*]quinolizinium derivatives **1a**–**d** (Figure 1), namely to apply the alternative base-free Suzuki–Miyaura coupling reaction [39–42] between the benzo[*b*]quinolizinium-9-trifluoroborate (**3b**) and aryldiazonium salts. We focused our attention on derivatives **1a**–**d** because in these cases a direct comparison with the already reported synthesis with a Suzuki–Miyaura reaction is possible. As we are particularly interested in benzo[*b*]quinolizinium derivatives with a large extension of the π-system, which should provide promising properties as G4-DNA ligands, we also focused our attention on the Sonogashira reaction as synthetic route to arylalkynyl-substituted derivatives. In this case, we aimed at donor-substituted derivatives such as **2b**–**d** since they were proposed to have ideal photophysical and DNA-binding properties. Herein, we present the successful Suzuki–Miyaura and Sonogashira coupling reactions of benzo[*b*]quinolizinium substrates. In addition, the absorption and emission properties of the novel arylalkynylbenzo[*b*]quinolizinium derivatives **2a**–**d** are reported (Figure 1), along with preliminary studies of their duplex and quadruplex DNA-binding properties.

## Results

### Synthesis

#### Synthesis of 9-aryl-substituted benzo[*b*]quinolizinium derivatives **1a–d**

**Scheme 1:**
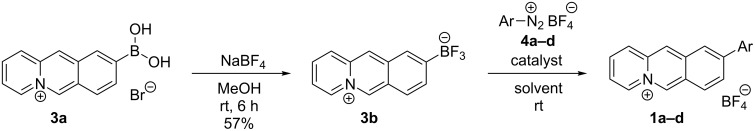
Synthesis of benzo[*b*]quinolizinium-9-trifluoroborate (**3b**) and 9-arylbenzo[*b*]quinolizinium derivatives **1a**–**d** (see Table 1 for assignment of indices **a**–**d** and reaction conditions).

The 9-aryl-substituted benzo[*b*]quinolizinium derivatives **1a–d** were prepared under base-free conditions by the Pd-catalyzed Suzuki–Miyaura reaction of the aryldiazonium salts **4a–d** with benzo[*b*]quinolizinium-9-trifluoroborate (**3b**). The latter substrate was obtained as analytically pure product in moderate yield by the reaction of benzo[*b*]quinolizinium-9-boronic acid (**3a**) [34] with NaBF_4_ (Scheme 1).

To identify appropriate reaction conditions for the base-free synthesis of derivatives 1**a**–**d**, different catalysts and solvents were tested for the cross-coupling reaction of benzo[*b*]quinolizinium-9-trifluoroborate (**3b**) and benzenediazonium salt **4a** (Scheme 1, Table 1). With Pd(dppf)_2_Cl_2_·CH_2_Cl_2_ or Pd(PPh_3_)_4_ as catalyst, no conversion was observed, whereas the reaction could be achieved with Pd(OAc)_2_ as a catalyst and water as a solvent (Table 1, entries 1–5). Thus, the latter reaction conditions were used for the synthesis of 9-arylbenzo[*b*]quinolizinium derivatives **1b**–**d** (Scheme 1, Table 1). The methoxyphenyl- and dimethylaminophenyl-substituted derivatives **1b** and **1c** were obtained in a moderate to good yield, but only trace amounts of the pyridyl-substituted derivative **1d** were formed as shown by the ^1^H NMR spectroscopic analysis of the reaction mixture (Table 1, entries 6–8). Nevertheless, the 9-pyridinyl derivative **1d** was obtained in low yield by the reaction of the trifluoroborate **3b** with the diazonium salt **4d** at 80 °C in DMF with Pd(PPh_3_)_4_ as a catalyst (Table 1, entry 9). It should be noted that some of these Suzuki–Miyaura coupling reactions require relatively long reaction times (Table 1, entries 6–9), which is a disadvantage considering the competing decomposition of the aryldiazonium ions under the reaction conditions. Thus, the corresponding diazonium salt was added in portions in intervals of 24 h until all of the substrate was consumed.

**Table 1:** Reaction conditions for the synthesis of 9-arylbenzo[*b*]quinolizinium derivatives **1a–d** according to Scheme 1.

Entry	Solvent	Catalyst	*t* (h)	Product	Yield (%)

1	H_2_O	Pd(OAc)_2_	48	**1a**, R = Ph	43
2	DME/H_2_O/MeOH	Pd(dppf)_2_Cl_2_·CH_2_Cl_2_^a^	24	**1a**, R = Ph	n.r.^b^
3	DMF	Pd(dppf)_2_Cl_2_·CH_2_Cl_2_^a^	24	**1a**, R = Ph	n.r.^b^
4	CH_3_CN	Pd(OAc)_2_	24	**1a**, R = Ph	n.r.^b^
5	CH_3_CN	Pd(PPh_3_)_4_	24	**1a**, R = Ph	n.r.^b^
6	H_2_O	Pd(OAc)_2_	168	**1b**, R = 4-MeO(C_6_H_5_)	95
7	H_2_O	Pd(OAc)_2_	144	**1c**, R = 4-Me_2_N(C_6_H_5_)	44
8	H_2_O	Pd(OAc)_2_	168	**1d**, 4-pyridyl	<2
9	DMF	Pd(PPh_3_)_4_	168	**1d**, 4-pyridyl	16

^a^dppf = 1,1’-bis(diphenylphosphino)ferrocene. ^b^No reaction.

#### Synthesis of 9-(arylethynyl)benzo[*b*]quinolizinium derivatives **2a–d**

**Scheme 2:**
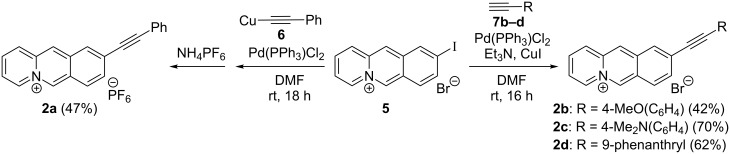
Synthesis of 9-(arylethynyl)benzo[*b*]quinolizinium derivatives **2a**–**d**.

The 9-(arylethynyl)benzo[*b*]quinolizinium derivatives **2a–d** were prepared by Pd-mediated Sonogashira coupling reactions of 9-iodobenzo[*b*]quinolizinium bromide (**5**) [43] with arylacetylene derivatives (Scheme 2). To suppress the ring opening of the benzo[*b*]quinolizinium ring by nucleophilic attack at the 6-position [34–35] two methods were used that avoid the addition or formation of strong nucleophiles during the reaction. In the first approach, (phenylethynyl)copper (**6**) [44] was prepared separately and subsequently made to react with the substrate **5** to provide derivative **2a** as hexafluorophosphate salt in moderate yield (Scheme 2). During the preparation of derivatives **2b**–**d** with this method a crude product was isolated that contains the desired compound along with unidentified impurities, as shown by ^1^H NMR spectroscopic analysis of the product. Unfortunately, the product could not be further purified.

In the second approach, the copper acetylide was formed in situ by the reaction of the acetylene derivative with triethylamine in the presence of Cu^+^ salts. Hence, the reaction of iodobenzo[*b*]quinolizinium **5** with arylacetylenes **7b**–**d** in the presence of one equivalent of triethylamine and CuI under anhydrous conditions gave (arylethynyl)benzo[*b*]quinolizinium derivatives **2b**–**d** in moderate to good yield (Scheme 2).

Several attempts to purify the derivatives **2a**–**d** by column chromatography failed. Apparently, these compounds decompose when in contact with the silica or alumina of the column, so that the pure products were only available by crystallization from appropriate solvents, which resulted in lower yields of these products.

#### Single crystal X-ray diffraction analysis of 9-(arylethynyl)benzo[*b*]quinolizinium derivatives **2a** and **2b**

**Figure 2:**
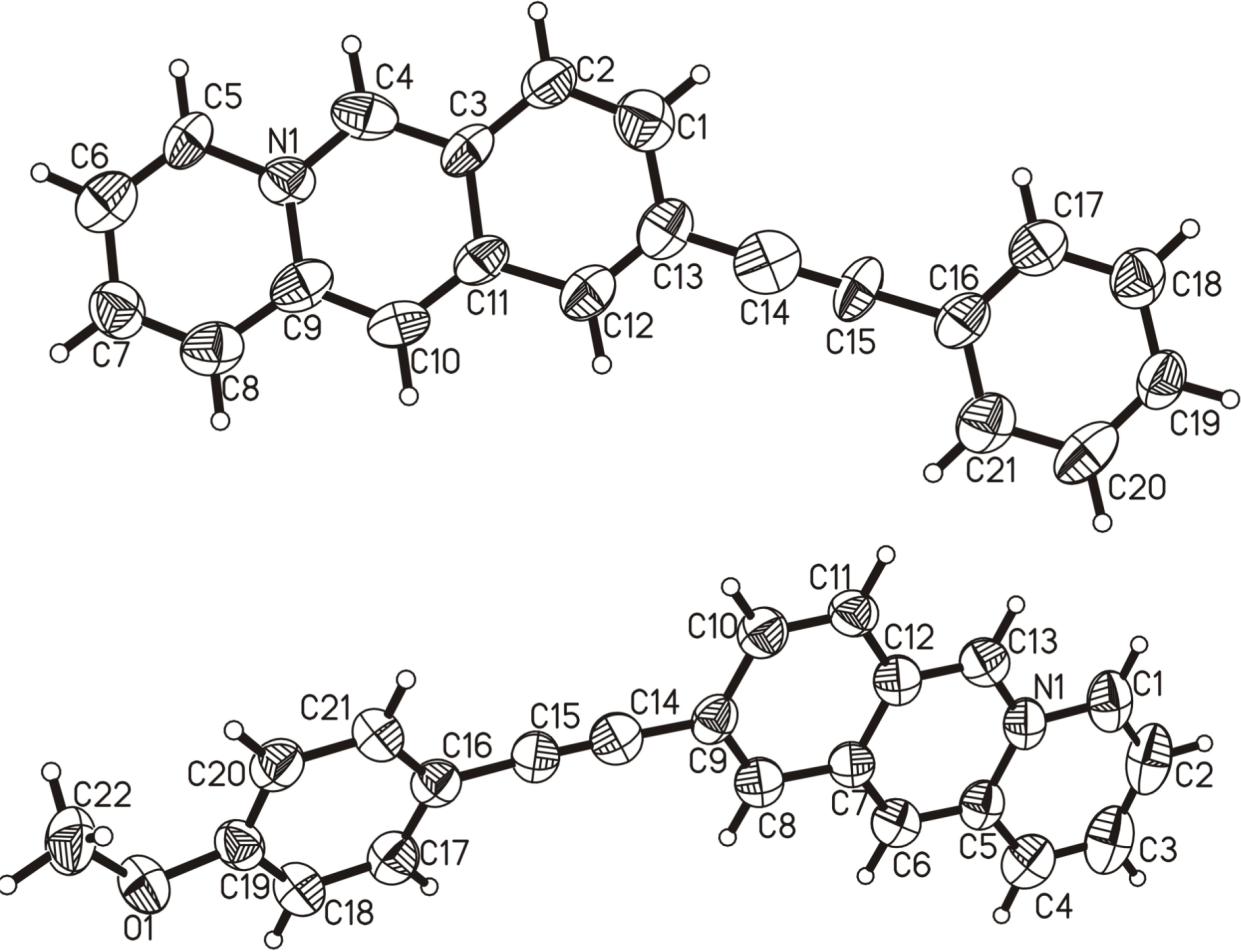
Molecular structures of derivatives **2a** (top) and **2b** (bottom) in the solid state. Ellipsoids are shown at the 50% probability level. The counter anions and solvent molecule were omitted for clarity.

Single crystals of derivatives **2a** and **2b** were obtained by crystallization from acetone and CHCl_3_/MeOH, respectively (Figure 2 and Figure S1, Supporting Information File 1). Derivative **2a** crystallizes in the triclinic space group 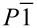 with two molecules in the unit cell. The crystals were twinned and the compound shows some considerable disorder. The chemical composition, however, was unanimously proven by the data. Derivative **2b** crystallizes with one molecule of CHCl_3_ as lattice solvent in the highly symmetric orthorhombic space group *I*2/*a* with 8 molecules in the unit cell. Both cations are essentially planar and π-stacked in an *anti*-head-to-tail (ht) arrangement in the solid state. A preference of such anti-ht arrangement was observed before in the crystal structures of two series of 9-substituted benzo[*b*]quinolizinium salts with halides or small alkyl substituents [45–46]. Stratford et al. attributed this observation to repulsion forces between the positively charged nitrogen atoms and π···π donor–acceptor attractions between the phenyl and pyridinium moieties. In our case, the situation is somehow more complex as the novel compounds bear aromatic substituents (via alkyne spacer) in the benzo[*b*]quinolizinium 9 position. These aromatic substituents now engage in π···π donor–acceptor attractions with the pyridinium moiety (outer most ring of the tricyclic moiety) and the two positively charged nitrogen atoms are per se much further apart due to the larger intramolecular separation between the intermolecularly interacting π-systems. In addition, the aromatic character of the substituent and its engagement in the π···π interaction also brings the two phenyl rings of adjacent benzo[*b*]quinolizinium moieties in close proximity, which can now also interact in an off-set π···π fashion. The contribution of the charge repulsion has, hence, to be less significant here and the preference for the anti-ht arrangement must be dominated by the π···π attractions. Centroid distances between the aromatic 9-substituent and the pyridinium moiety are 3.619 Å for **2a** (C16 → C21; C5 → C9, N1) and 3.676 Å for **2b** (C16 → C21; C1 → C5, N1), respectively (Figure S1, Supporting Information File 1). These separations are comparably short as the reported ones range from 3.69 Å to 3.99 Å [46]. The π-system separation between the centroids of the two benzo[*b*]quinolizinium phenyl rings are 3.803 Å for **2a** (C1, C2, C3, C11, C12, C13) and 3.599 Å for **2b** (C7 → C12), respectively. Notably, for **2b** the phenyl to phenyl π···π interaction of one molecule is not with the same neighbor as the π···π donor–acceptor attraction between the phenyl and pyridinium rings. Therefore, these two distinct π-system-based attractions alternate and form infinite chains of molecules roughly protruding along the a axis. In **2a** both interactions are with the same neighbor leading to distinct dimeric associates.

In the individual molecules, the C–C bond lengths of the alkyne unit are 1.24 Å (C14–C15) for the triple bond and 1.41 Å (C13–C14 and C15–C16) for the single bonds in compound **2a**, while in derivative **2b** they are 1.18 Å (C14–C15) and 1.45 Å (C9–C14 and C15–C16), respectively. Moreover, the π-surface of derivative **2b** deviates slightly more from the mean plane as compared with **2a**, i.e., as the torsion angle C8–C9–C16–C17 is −12.0° whereas it is 5.6° (C12–C13–C16–C21) in **2a**. These data indicate a slightly more pronounced delocalization of π-electrons within the diarylalkyne unit of compound **2a**, at least in the solid state.

#### Absorption and emission properties of 9-(arylethynyl)benzo[*b*]quinolizinium derivatives **2a–d**

**Figure 3:**
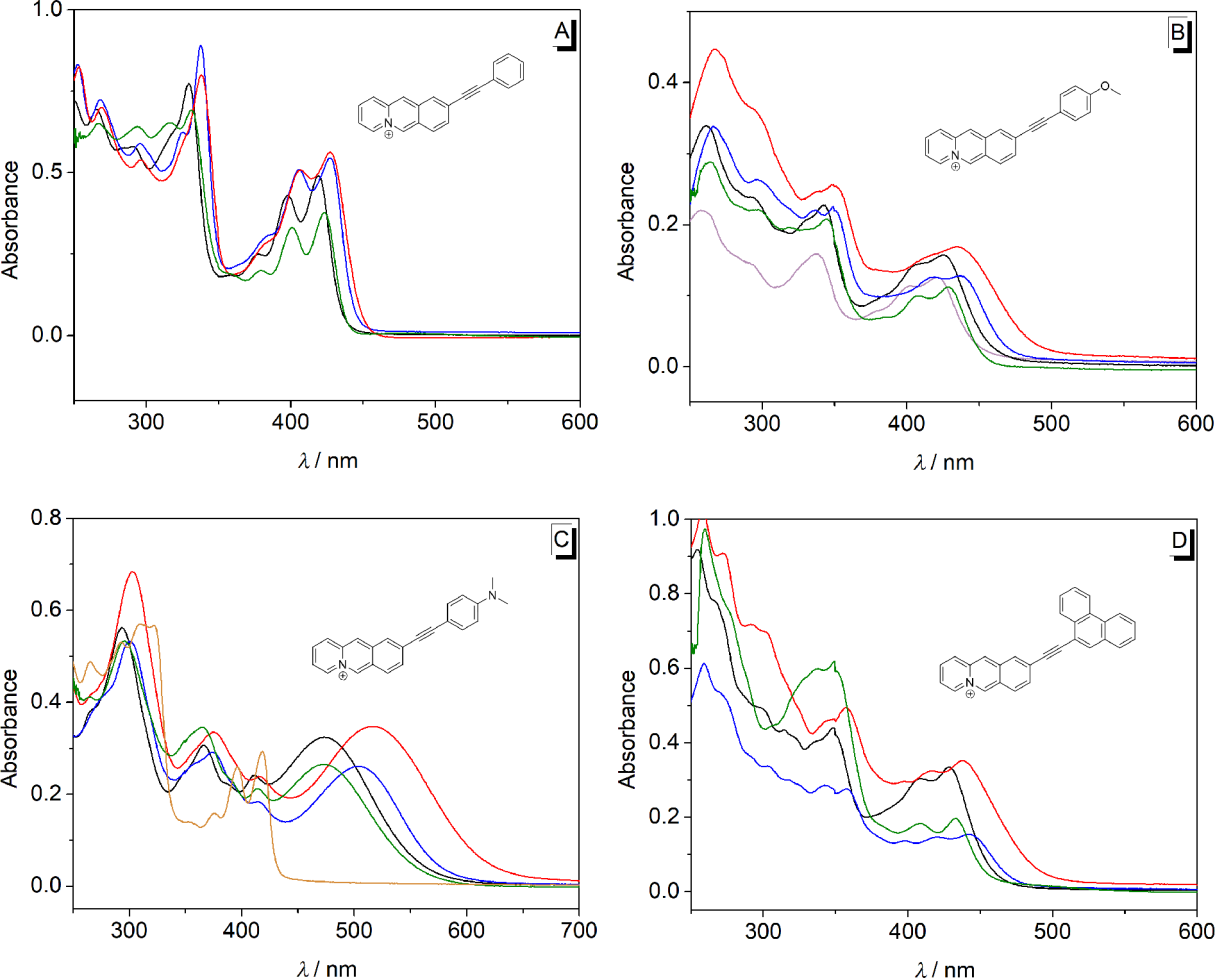
Absorption spectra of derivatives **2a** (A), **2b** (B), **2c** (C) and, **2d** (D); *c* = 20 μM; solvents: H_2_O (magenta), MeOH (black), CHCl_3_ (blue), CH_2_Cl_2_ (red), DMSO (green) and 1 N HCl (orange).

In general, compounds **2a–d** have a low solubility in water and derivative **2d** is moderately soluble in DMSO. The absorption spectra of 9-(arylethynyl)benzo[*b*]quinolizinium derivatives **2a**,**b**,**d** show two low-energy maxima between 380 nm and 450 nm which resemble the ones of similar aryl-substituted benzo[*b*]quinolizinium [34] and naphthoquinolizinium [36] derivatives (Figure 3, Table 2). As a notable exception, the derivative **2c** has a broad absorption band with maximum wavelength depending on the solvent, namely at 470 nm in MeOH, 515 nm in CH_2_Cl_2_ and 505 nm in CHCl_3_ (Figure 3C). At low pH, the broad long wavelength absorption band of **2c** disappeared and a new absorption band was formed (λ_max_ = 418 nm) that is similar to that of the parent compound **2a** (Figure 3C). It should be noted that the benzo[*b*]quinolizinium derivatives **2a–d** have lower absorbance and significantly broadened spectra in less polar solvents, presumably due to their low solubility and the resulting aggregation in these media.

**Table 2:** Absorption and emission properties of benzo[*b*]quinolizinium derivatives **2a**–**d**.

	**2a**			**2b**		**2c**		**2d**		
Solvent^a^	λ_abs_^b^ / nm	λ_fl_^c^ / nm	Φ_fl_^d^ / 10^−2^	λ_abs_^b^ / nm	λ_fl_^c^ / nm	λ_abs_^b^ / nm	λ_fl_^c^ / nm	λ_abs_^b^ / nm	λ_fl_^c^ / nm	Φ_fl_^d^ / 10^−2^

H_2_O	419	460	< 1	422	562	434	n.d.^e^	422	572	<1
MeOH	419	462	64	426	545	470	n.d.^e^	428	571	6
EtOH	420	462	39	428	558	481	n.d.^e^	430	570	6
MeCN	418	460	40	423	n.d.^e^	472	n.d.^e^	428	578	5
DMSO	423	n.d.^e^	n.d.^e^	428	n.d.^e^	473	n.d.^e^	432	570	<1
aceton	419	460	34	424	n.d.^e^	472	n.d.^e^	429	580	2
CH_2_Cl_2_	428	470	44	435	554	515	497	439	560	4
CHCl_3_	427	470	43	438	485	505	501	443	460	2

^a^Solvents in order of decreasing *E*_T_ values [47]. ^b^Long-wavelength absorption maximum; *c* = 20 μM. ^c^Fluorescence emission maximum (Abs. = 0.10 at excitation wavelength); λ_ex_ = 375 nm. ^d^Fluorescence quantum yield relative to coumarin 1 [47–48]; estimated error for Φ_fl_: ± 10%. ^e^Not determined.

**Figure 4:**
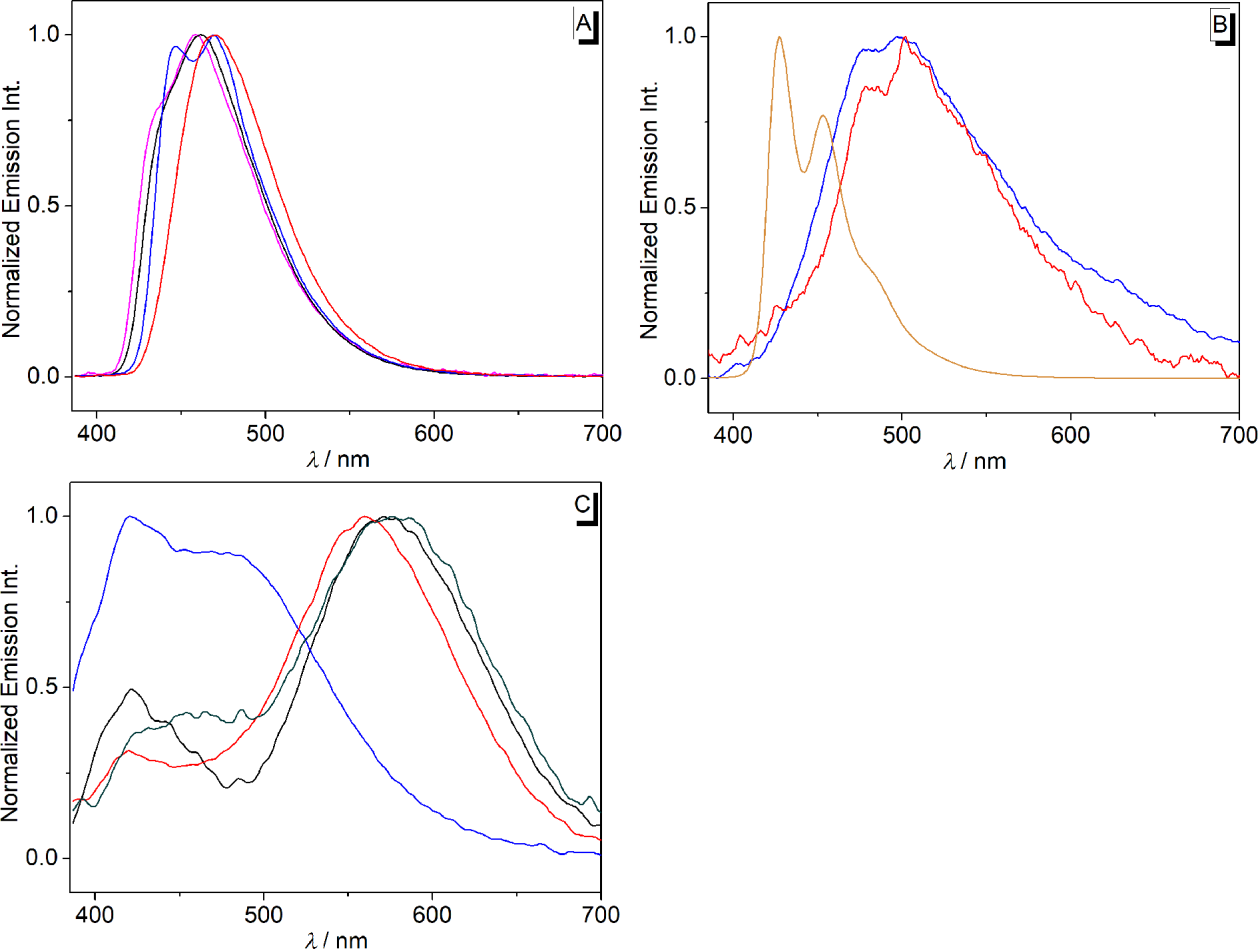
Emission spectra of derivatives **2a** (A), **2c** (B) and **2d** (C); *c* = 20 μM; λ_ex_ = 375 nm; solvents: H_2_O (magenta), MeOH (black), CHCl_3_ (blue), CH_2_Cl_2_ (red), DMSO (green) and 1 N HCl (orange); λ_ex_ = 375 nm.

Except for the derivative **2a** the arylethynylbenzoquinolizinium derivatives have low emission quantum yields (Table 2, Figure 4). The derivative **2a** has a moderate to high fluorescence intensity with slight deviations of the emission maxima in different solvents (Table 2, Figure 4A). In chloroform, it has two emission maxima at 446 and 470 nm. The derivative **2d** has a weak fluorescence intensity in different solvents (Φ_fl_: 0.02–0.06). In chloroform, it shows an emission maximum at 460 nm, while in other solvents it has emission maxima between 560 and 580 nm with a shoulder at 430 nm (Figure 4C). On the other hand, derivatives **2b** and **2c** exhibit very weak fluorescence intensity in different solvents (Φ_fl_ < 0.02). Derivative **2c** shows only a weak emission (Φ_fl_ = 0.02) in 1 N HCl with significantly blue-shifted emission maxima at 427 and 454 nm (Figure 4B).

**Figure 5:**
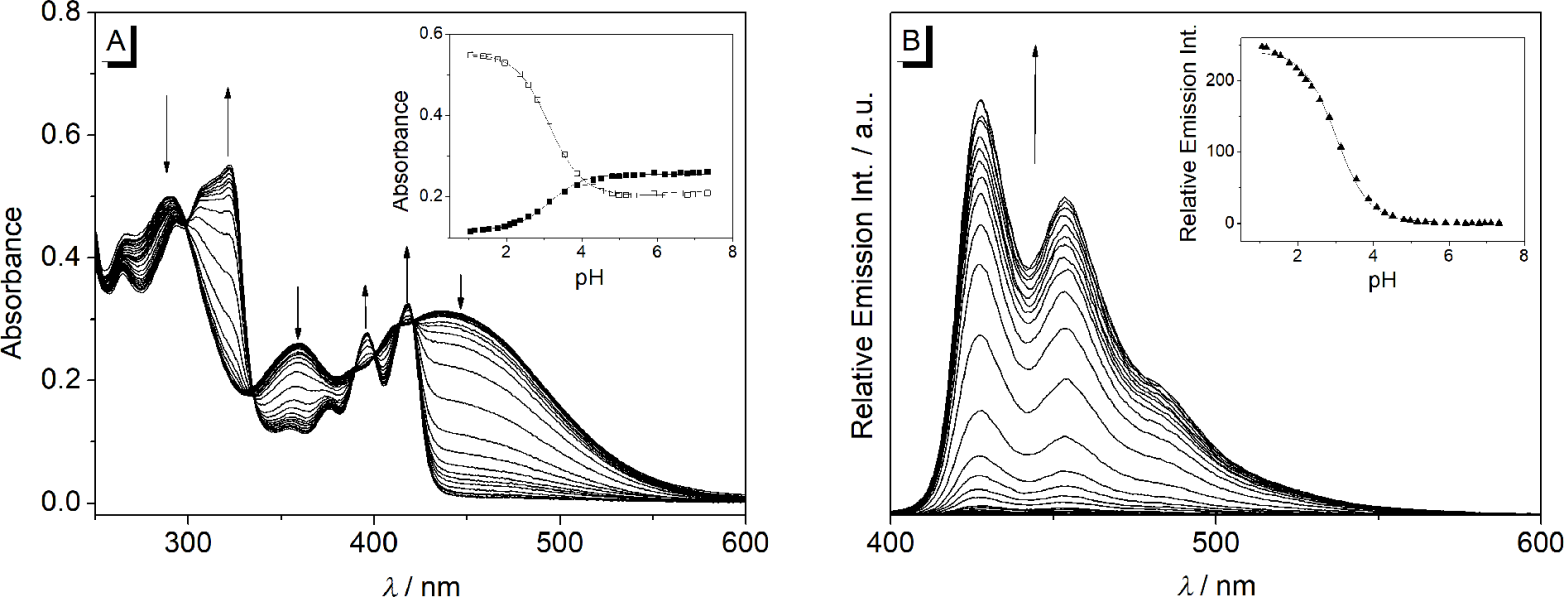
Photometric (A) and fluorimetric (B) acid-base titration of **2c**; *c* = 20 μM in Britton–Robinson buffer; λ_ex_ = 375 nm. Arrows indicate the development of absorption or emission bands with decreasing pH value. Inset: Plot of the absorption (A) at 360 nm (black rectangle) and 322 nm (white rectangle) and emission (B) at 427 nm (black triangle) versus pH. Lines denote the best fit of experimental data to the theoretical model.

To further assess the effect of the pH on the absorption and emission properties of derivative **2c**, photometric and fluorimetric acid–base titrations of **2c** were performed (Figure 5). With decreasing pH of the solution (pH 7.3–1.1), new absorption bands developed at λ_max_ = 418 nm, 395 nm and 322 nm, along with the disappearance of the initial broad long wavelength absorption (Figure 5A). The emission intensity of derivative **2c** increased by a factor of 250 with decreasing pH value (Figure 5B). The p*K*_a_ value of the protonated amine **2c** in water was determined from the titration curve to be 3.1 which is in the same range as the ones of 9-(*p*-amino)phenylacridinium ions (p*K*_a_ = 2.5–3.5) [49–50] and the dimethylaminophenyl-substituted benzo[b]quinolizinium ion [34] (p*K*_a_ = 3.8).

#### Photometric and fluorimetric DNA titrations of 9-(arylethynyl)benzo[*b*]quinolizinium derivatives **2a–d**

**Figure 6:**
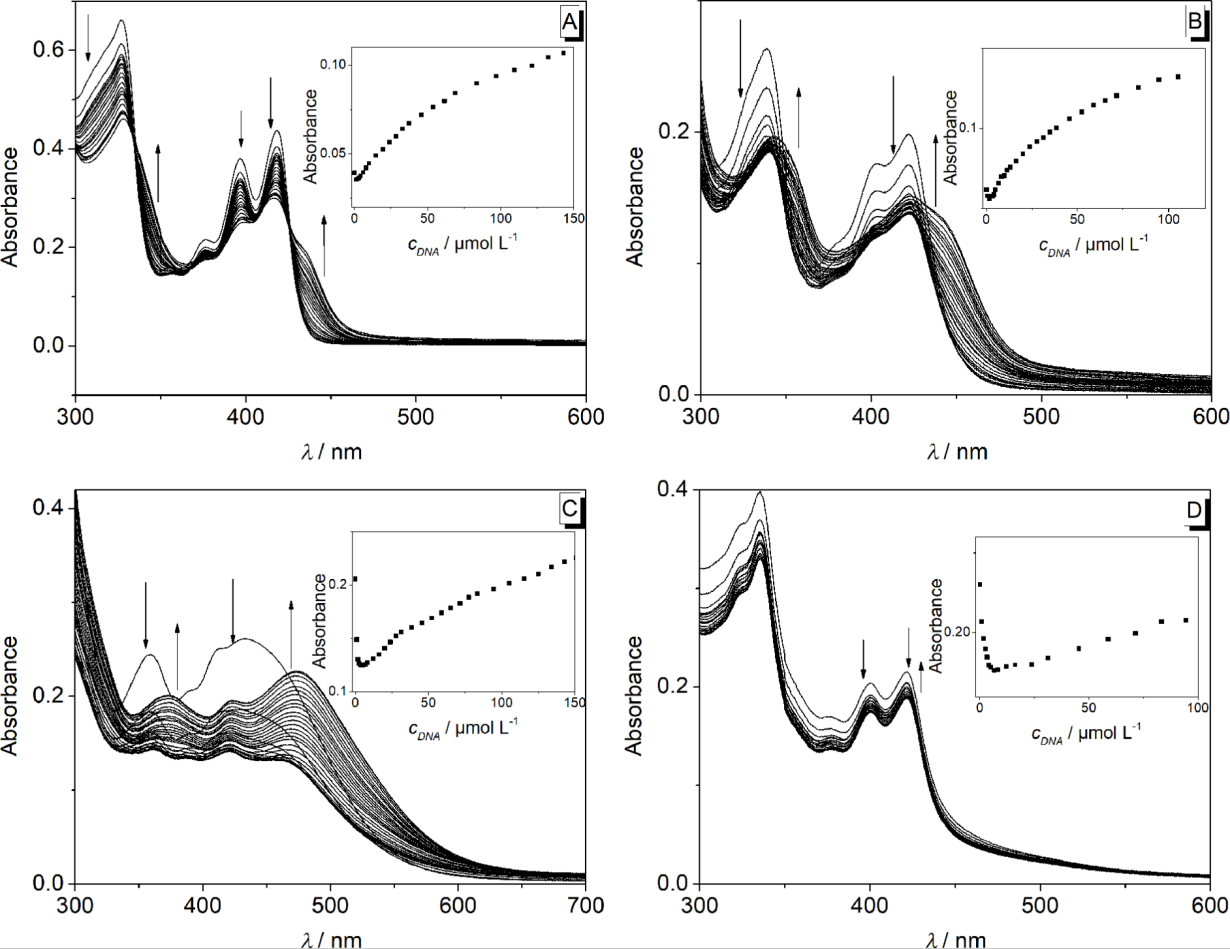
Photometric titration of **2a** (A), **2b** (B), **2c** (C), and **2d** (D) with ct DNA in BPE buffer (16 mM Na^+^; 5% DMSO; pH 7.0); *c***_L_** = 20.0 μM. Arrows indicate the development of bands with increasing DNA concentration. Inset: Plot of the absorption at long wavelength versus DNA concentration.

**Figure 7:**
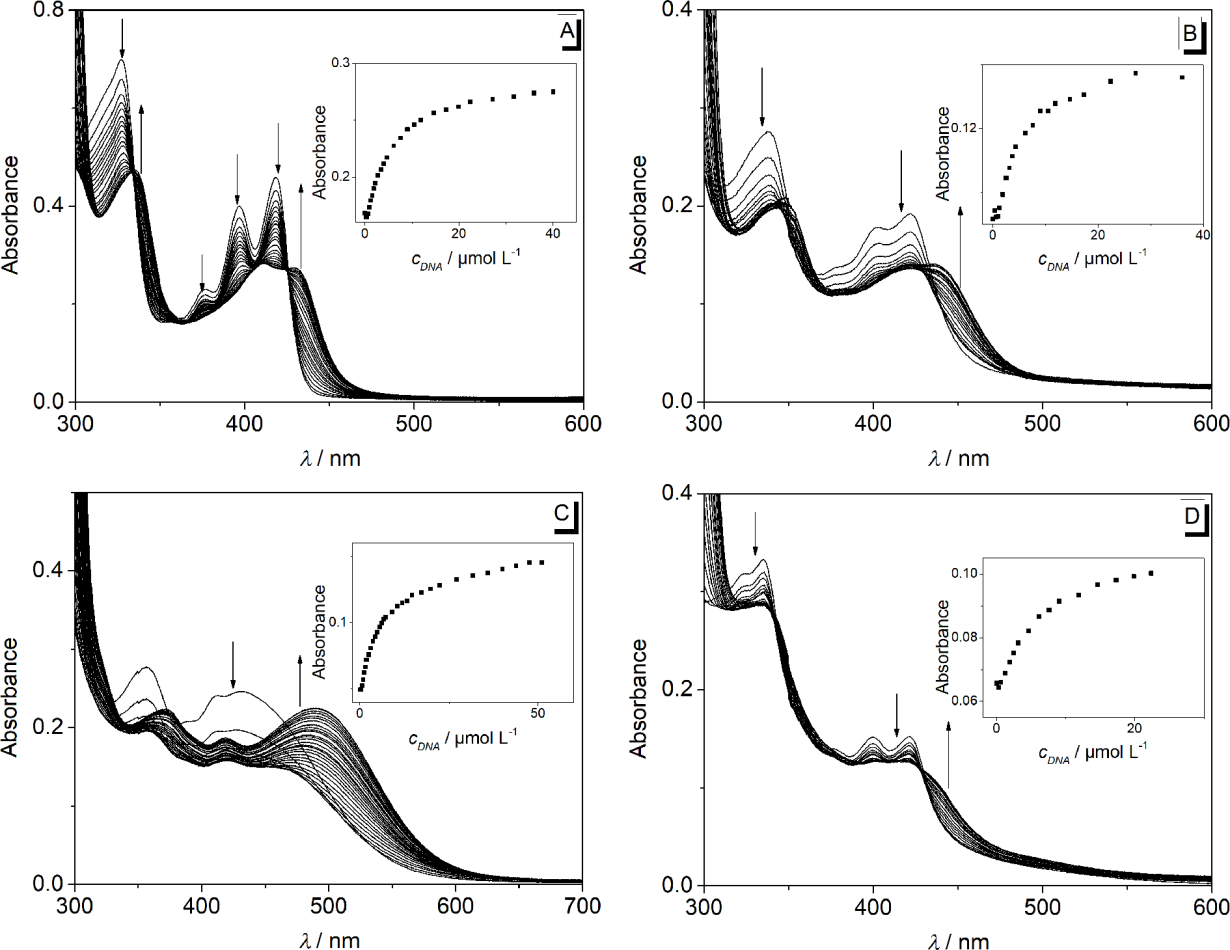
Photometric titration of **2a** (A), **2b** (B), **2c** (C) and **2d** (D) with **22AG** in potassium phosphate buffer (95 mM K^+^; 5% DMSO; pH 7.0); *c***_L_** = 20.0 μM. Arrows indicate the development of bands with increasing DNA concentration. Inset: Plot of the absorption at long wavelength versus DNA concentration.

**Figure 8:**
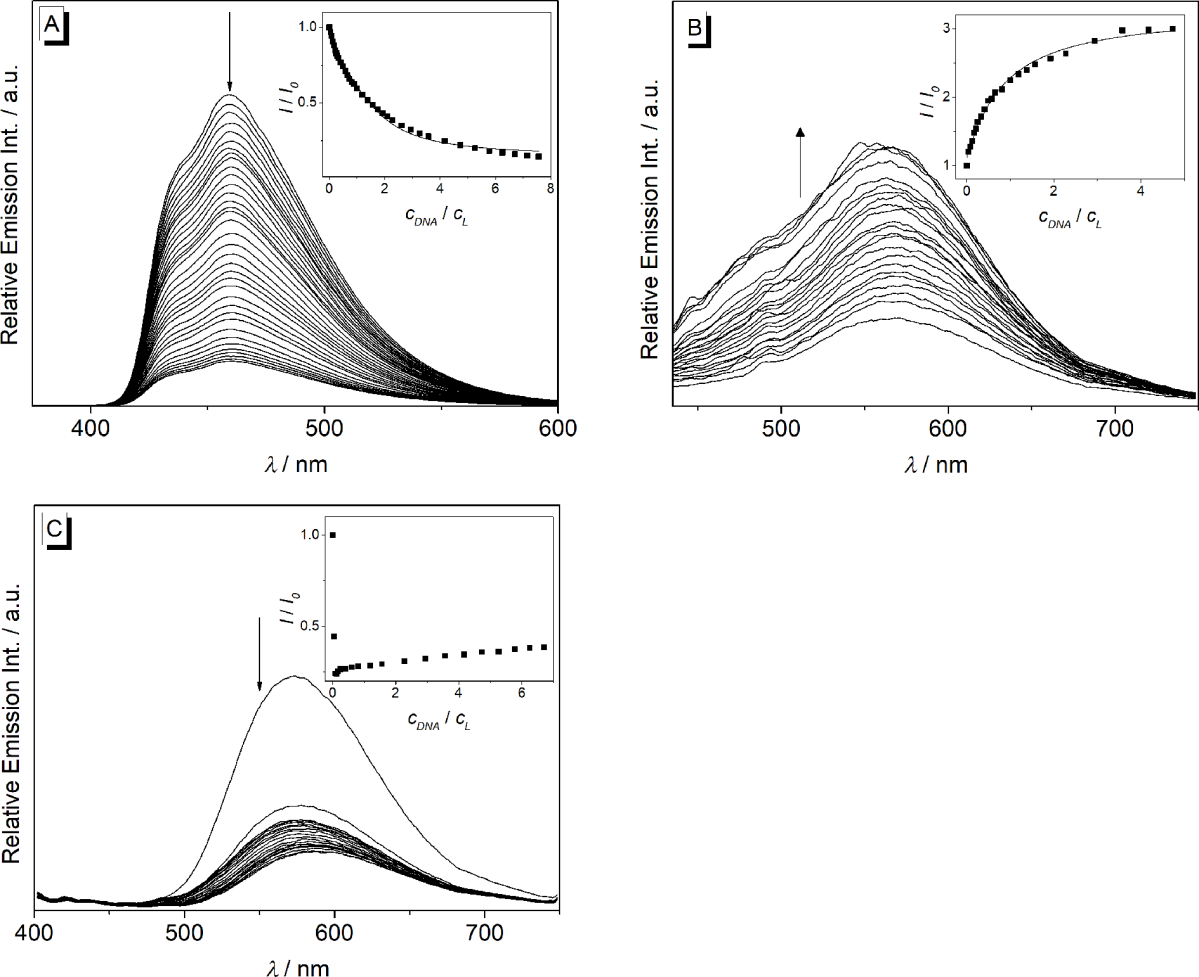
Fluorimetric titration of **2a** (A), **2b** (B) and **2d** (C) with ct DNA in potassium phosphate buffer (95 mM K^+^; 5% DMSO; pH 7.0); *c*_Ligand_ = 20.0 μM. Arrows indicate the development of the bands with increasing DNA concentration. Inset: Plot of the relative emission intensity, *I*/*I*_0_ *versus c*_DNA_/*c*_L_. Lines denote the best fit of experimental data to the theoretical model; λ_ex_ = 335 nm (A), 420 nm (B) and 380 nm (C).

**Figure 9:**
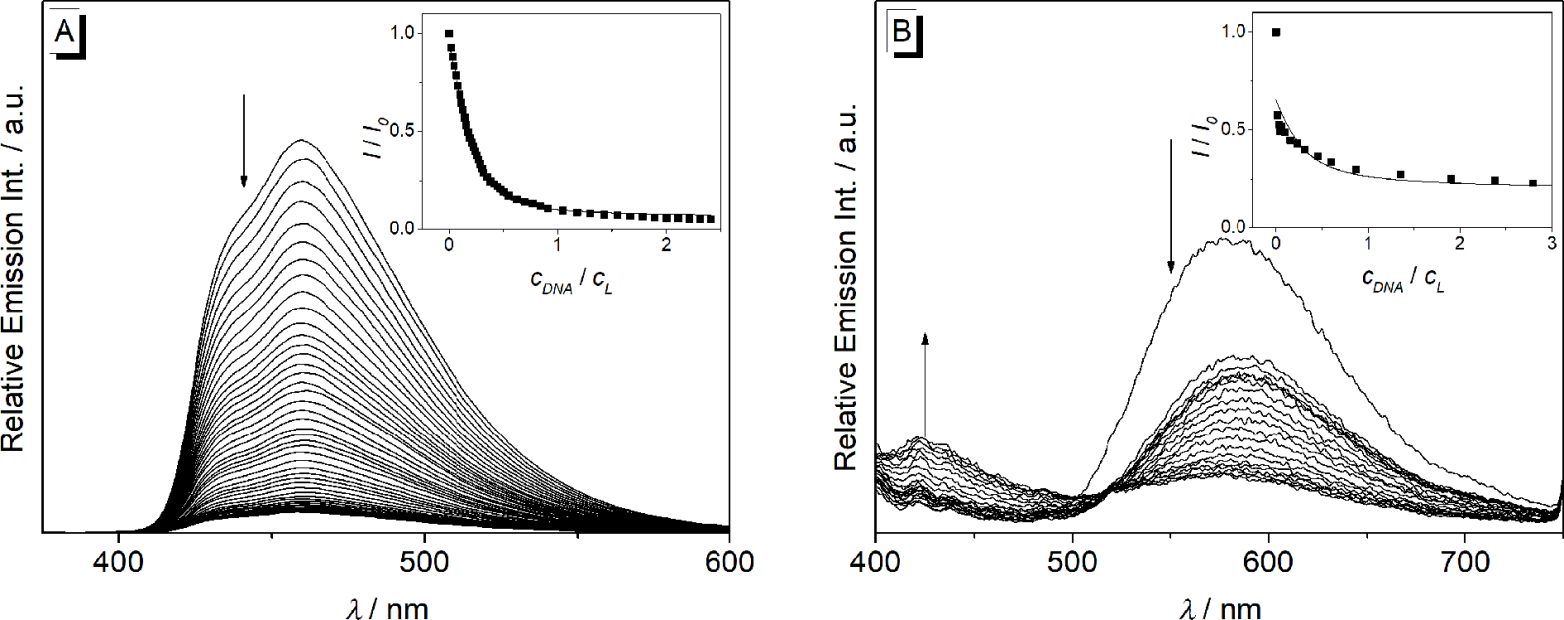
Fluorimetric titration of **2a** (A) and **2d** (B) with **22AG** in potassium phosphate buffer (95 mM K^+^; 5% DMSO; pH 7.0); *c*_Ligand_ = 20.0 μM. Arrows indicate the development of the bands with increasing DNA concentration. Inset: Plot of the relative emission intensity, *I*/*I*_0_ *versus c*_DNA_/*c*_L_. Lines denote the best fit of experimental data to the theoretical model; λ_ex_ = 335 nm (A) and 380 nm (B).

The interactions of the arylethynylbenzoquinolizinium derivatives **2a**–**d** with ct DNA and G4-DNA **22AG** [d(AG_3_T_2_AG_3_T_2_AG_3_T_2_AG_3_)] were investigated with photometric and fluorimetric titrations (Figures 6–9, Table 3). In general, a hypochromic effect and a bathochromic shift were observed by the addition of DNA. For example, the addition of ct DNA and G4-DNA **22AG** to derivative **2a** led to the evolution of a new maximum at 437 nm and 423 nm, respectively, with an isosbestic point at 325 nm. However, during the titration of DNA to the derivatives **2b–d** isosbestic points were not formed. In the case of **2d**, only a hypochromic effect was observed upon the addition of ct DNA (Figure 6D). In contrast, the addition of **22AG** to **2d** resulted in a red shift with Δλ_abs_ = 16 nm. Notably, the addition of DNA to derivative **2c** led to the largest bathochromic shifts and hypochromic effect (ct DNA: Δλ_abs_ = 42 nm; **22AG**: Δλ_abs_ = 58 nm). Only the data extracted from the photometric titration of **22AG** to derivatives **2b** and **2c** could be used to deduce the binding constant *K*_b_ (Figure S2, Table 3).

**Table 3:** Absorption and emission properties of ligands **2a–d** upon the addition of DNA, and binding constants *K_b_*.

Ligand	ct DNA				**22AG**			
	λ_abs_^a^ / nm	Δλ_abs_^b^ / nm	*I*/*I*_0_^c^	*K*_b_^d^ / 10^4^ M^−1^	λ_abs_^a^ / nm	Δλ_abs_^b^ / nm	*I*/*I*_0_^c^	*K*_b_^d^ / 10^4^ M^−1^

**2a**	437	18	0.14	14	432	19	0.05	22
**2b**	443	21	3	1.5	440	18	n.d.^e^	2.6^f^
**2c**	476	42	n.d.^e^	n.d.^e^	492	58	n.d.^e^	1.6^f^
**2d**	422	0	0.38	n.d.^e^	438	16	0.19	3.0

^a^Long-wavelength absorption maximum of the DNA-bound ligand. ^b^Shift of the long-wavelength absorption maximum between free and bound ligand. ^c^Relative emission intensity, *I*/*I*_0_ (*I* = emission intensity of DNA-bound ligand at saturation, *I*_0_ = emission of unbound ligand). ^d^Binding constant of ligand–DNA complex, *K_b_*, determined from fluorimetric titrations. ^e^Not determined. ^f^*K*_b_ determined from photometric titrations; DNA concentration in base pairs for ct DNA and in oligonucleotide for **22AG**.

The addition of ct DNA to the derivative **2a** led to quenching of the emission intensity (Figure 8A). In contrast, a light-up effect with a factor of 3 was observed upon the addition of ct DNA to derivative **2b** (Figure 8B, Table 2). Notably, the emission intensity of derivative **2d** at λ_fl_ = 572 nm decreased at the beginning of the titration with ct DNA at a ligand–DNA ratio (*LDR*) > 8. With further addition of ct DNA, however, the emission intensity increased slightly at the same emission wavelength (Figure 8C). The binding constants, *K*_b_, between ct DNA and derivatives **2a** (1.4 × 10^5^ M^−1^) and **2b** (1.5 × 10^4^ M^−1^) were determined from the fluorimetric titrations by fitting the resulting binding isotherms to the theoretical model (insets in Figure 8, Table 3) [51]. Unfortunately, the data obtained from the fluorimetric titration of **2d** with ct DNA could not be fitted to the theoretical model. The low emission intensity of the derivative **2c** was not affected by the addition of ct DNA or **22AG**.

The emission intensity of **2a** was quenched upon addition of G4-DNA **22AG** (Figure 9A). Remarkably, the addition of **22AG** to derivative **2d** resulted in a decrease of the emission intensity at λ_fl_ = 572 nm and a new weak emission band evolved at λ_fl_ = 425 nm. The emission intensity of **2b** was not influenced significantly by the addition of **22AG**. The binding constants *K*_b_ between **22AG** and derivatives **2a** (2.2 × 10^5^ M^−1^) and **2d** (3.0 × 10^4^ M^−1^) were determined from the fluorimetric data by fitting the binding isotherms to the theoretical model (insets in Figure 9, Table 2) [51].

## Discussion

### Pd-mediated coupling reactions of halogenobenzo[*b*]quinolizinium derivatives

Although it was shown in this work that in particular cases appropriately substituted benzo[*b*]quinolizinium substrates can be functionalized as aryl- or alkynyl-substituted derivatives by Sonogashira and base-free Suzuki–Miyaura coupling reactions, it is obvious that this synthetic approach has its limitations. As compared with the corresponding quinolizinium substrates, that can be used for a variety of metal-mediated coupling reactions [28–32], the benzo[*b*]quinolizinium core appears to be very sensitive towards the reaction conditions, leading to serious side or secondary reactions. All experimental results indicate that the "usual" experimental protocols cannot be applied due to the high susceptibility of the benzo[*b*]quinolizinium ring towards nucleophilic attack at 6-position that leads to ring opening [37–38]. Thus, the Sonogashira reaction of **5** requires either the separate generation of copper acetylide or strict water-free conditions to avoid the formation of hydroxide ions. To avoid the potential interference of bases, we attempted to improve of the conditions for the Suzuki–Miyaura coupling in the base-free variant using aryldiazonium reagents [39]. Although the coupling reactions between aryldiazonium salts and arylboronic acids or esters with base-free conditions are known [39,42], in our hands the reaction of benzo[*b*]quinolizinium-9-boronic acid (**3a**) with benzenediazonium tetrafluoroborate **4a** only resulted in the formation of the benzo[*b*]quinolizinium-9-trifluoroborate (**3b**). Consequently, we used the latter substrate for subsequent synthesis, as it has been reported that organotrifluoroborates may also be employed as starting materials in Suzuki–Miyaura coupling reactions of aryl halides [52–53]. Indeed, starting from benzo[*b*]quinolizinium-9-trifluoroborate (**3b**) and the corresponding aryldiazonium ions the 9-arylbenzo[*b*]quinolizinium derivatives **1a–d** were available in yields that are comparable, or even slightly higher, than the ones obtained with the Suzuki–Miyaura reaction of benzo[*b*]quinolizinium-9-boronic acid (**3a**) with bromoarenes [34].

**Scheme 3:**
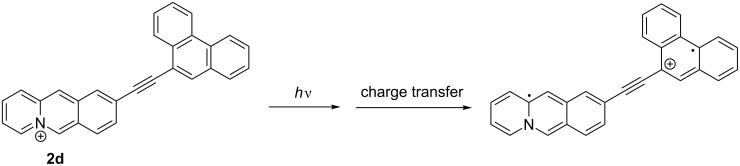
Photoinduced charge transfer upon the excitation of derivative **2d**.

In our previous attempts to synthesize the corresponding benzo[*b*]quinolizinium-9-trifluoroborate (**3b**), the reaction of benzo[*b*]quinolizinium-9-boronic acid (**3a**) with KHF_2_ only resulted in a partly contaminated product [34]. In this work, we used NaBF_4_ as reagent, as we have rather accidentally observed that it can be used for the synthesis of the trifluoroborate **3b** (see above); however, with lower yield (Scheme 3). Interestingly, to the best of our knowledge, there is only one report in the literature about the explicit use of NaBF_4_ as fluorinating reagent for boronic acids [54], and we have not investigated the general applicability of this reaction so far. Nevertheless, this approach appears to be a useful, complementary method to the usual fluorination with KHF_2_. And it may be suggested that this simple procedure might be used as a general straightforward method for the generation of the synthetically highly useful aryltrifluoroborates.

### Absorption and emission properties of 9-(arylethynyl)benzo[*b*]quinolizinium derivatives

The absorption maxima of the 9-(arylethynyl)benzo[*b*]quinolizinium derivatives **2a**–**c** are slightly red-shifted as compared to the corresponding 9-arylbenzo[*b*]quinolizinium compounds **1a**–**c** [34]. And in similar analogy, the extent of the red shift (H < OMe < NMe_2_) corresponds well with the strength of the donor–acceptor interplay between the electron-donating aryl substituent and the benzo[*b*]quinolizinium chromophore. The absorption properties depend only slightly on the solvent properties indicating that the corresponding ground state and vertical excited states are stabilized by the solvents to the same degree. As the only exception, larger red shifts of the absorption maxima of derivatives **2a**–**d** were observed in CHCl_3_ and CH_2_Cl_2_, which is presumably caused by the high polarizability of these solvents, as frequently observed with cationic dyes [34,46,55–56]. In the case of alkaline compound **2c**, the protonation of the amino group changes the ammonium-substituted aryl substituent to an electron acceptor which leads to a blue shift of the absorption maxima (Figure 3C).

The emission properties of the phenyl-substituted derivative **2a** do not depend significantly on the solvent properties, which indicate the absence of specific stabilization or destabilization of the excited molecule, even after solvent relaxation. In contrast, the phenanthryl-substituted derivative **2d** shows fluorosolvatochromism, specifically indicated by the strong blue shift in CHCl_3_. This effect is presumably caused by a charge shift (CS) or, more likely, by a charge transfer (CT) in the excited state from the electron-donating aryl unit to the excited quinolizinium (Scheme 3) [57], which has been proposed also to take place in structurally resembling excited biaryl-type acridinium, benzo[*b*]quinolizinium and naphtho[*b*]quinolizinium derivatives [36,58–64]. The CS/CT leads to an intermediate excited molecule with a charge neutral quinolizinyl radical and the radical cation of the phenanthryl unit (Scheme 3) that is well stabilized in polar solvents after solvent relaxation. At the same time, less polar solvents such as CHCl_3_ cannot stabilize this intermediate so that emission occurs from the energetically higher first local excited (LE) state which results in the blue-shifted emission. It should be noted that this blue-shifted emission band was also observed in polar solvents though with less intensity (Figure 4), which indicates that the emission from the LC state can compete with the charge shift and solvent relaxation, leading to dual emission.

Remarkably, the emission quantum yields of the methoxy- and amino-substituted derivatives **2b** and **2c** are very low (Φ_fl_ < 0.02). Such low emission intensities have been observed also for donor-substituted 9-arylbenzo[*b*]quinolizinium derivatives and explained either with a radiationless deactivation of the excited state by torsional relaxation or by a photoinduced electron transfer [33,49,65–66]. The effect of the donor substituent on the emission quenching was supported by the strong increase of the emission quantum yield of **2c** upon protonation of the amino group, that is, by the transformation of the donor to an acceptor substituent (Figure 5) [34]. Considering the water solubility of compound **2c**, though just moderate, the emission light-up effect may be used for fluorimetric detection of slightly acidic aqueous media.

### Interactions with DNA

The spectrometric titrations of DNA to compounds **2a**–**d** revealed the characteristic spectroscopic features of ligand–DNA interactions, namely a hypochromic effect and red shift of the absorption bands as well as emission quenching or enhancement upon addition of the nucleic acid. Moreover, the binding constants *K*_b_, as determined from the resulting binding isotherms, are in the same range (*K*_b_ = 2.0–22 × 10^4^ M^−1^, Table 3) of known DNA-intercalating benzo[*b*]quinolizinium derivatives [52,67–68], so that it may be concluded that the derivatives **2a**–**d** bind to DNA in a similar binding mode. Notably, a pronounced decrease of the long-wavelength absorption followed by the development of new band at longer wavelength was observed during the photometric titrations (Figure 6 and Figure 7), and only in the titration of the phenylethynyl-substituted derivative **2a** an isosbestic point was formed. These observations clearly show that the ligands bind in at least two different binding modes to DNA. Considering the low solubility of these compounds in water it is assumed that at the beginning of the titration, i.e., with large ligand–DNA ratio and a paucity of DNA binding sites, the ligand forms aggregates along the DNA backbone. With increasing DNA concentration more binding sites are available such that the ligands can intercalate. In the case of quadruplex DNA, the derivatives **2a**–**d** show a typical titration signature for ligands that bind to the quadruplex by terminal π-stacking [14]; however, in analogy to the binding to duplex DNA the derivatives **2b**–**d** form aggregates along the DNA backbone at large ligand–DNA ratio, i.e., at the beginning of the titration.

The fluorescence intensity of the derivatives **2a** and **2d** is significantly quenched by the addition of DNA, respectively (Figure 8 and Figure 9). This observation usually indicates a photoinduced electron transfer between the excited molecules and the DNA bases [69]. By contrast, the association of ct DNA with the methoxy-substituted derivative **2b** led to an increase of the low emission intensity by a factor of 3 (Figure 8B). Although this effect is rather small, it indicates the suppression of a deactivation pathway in the excited state upon the accommodation of **2b** in a constrained binding site of ct DNA, presumably due to the restriction of the conformational flexibility inside the binding site [65].

## Conclusion

In summary, different synthetic approaches toward the Pd-mediated coupling reactions of benzo[*b*]quinolizinium derivatives were assessed that enable the functionalization and further development of this useful class of compounds. In particular, we demonstrated that optimized base-free Suzuki–Miyaura and Sonogashira coupling reactions can be used for the synthesis of aryl- and arylalkynyl-substituted benzo[*b*]quinolizinium derivatives in moderate to good yields. Therefore, the optimized protocol for Pd-mediated reactions may be employed for other base-sensitive substrates as well.

The photophysical properties as well as the DNA-binding properties of the (arylethynyl)benzo[*b*]quinolizinium derivatives were studied. It was demonstrated that derivatives **2a–d** bind to duplex and quadruplex DNA with binding constants *K*_b_ of 0.2–2.2 × 10^5^ M^−1^. Unfortunately, a differentiation between duplex and quadruplex DNA by derivatives **2a–d** was not observed. Therefore, future work has to focus on further functionalizations that lead to selective binding of the ligands to particular DNA forms, e.g., by fine tuning of the stereoelectronic or steric properties of substituents.

## Supporting Information

File 1 Additional spectral data, detailed description of the experiments performed, ^1^H NMR of the derivatives **2a–d** and crystallographic data.
