# Supplementary material for: Synthesis of 9-arylalkynyl- and 9-aryl-substituted benzo[b]quinolizinium derivatives by Palladium-mediated cross-coupling reactions
Source: Beilstein J Org Chem. 2018 Jul 23;14:1871–84. doi: 10.3762/bjoc.14.161 (PMC6071731; doi:10.3762/bjoc.14.161)
Supplement: File 1 — Additional spectral data, detailed description of the experiments performed, 1H NMR of the derivatives 2a–d and crystallographic data. [file Beilstein_J_Org_Chem-14-1871-s001.pdf]

Supporting Information

for

Synthesis of 9-arylalkynyl- and 9-aryl-substituted benzo[*b*]quinolizinium derivatives by Palladium-mediated cross-coupling reactions

Siva Sankar Murthy Bandaru[1,2], Darinka Dzubiel[1], Heiko Ihmels[1,*], Mohebodin Karbasiyoun[1], Mohamed M. A. Mahmoud[1] and Carola Schulzke[2]

Address: [1]Department of Chemistry and Biology, University of Siegen, Siegen, Germany and [2]Institute of Biochemistry, University of Greifswald, Greifswald, Germany

Author names are in alphabetical order that does not reflect the specific contribution of each author.

Email: Heiko Ihmels - ihmels@chemie.uni-siegen.de

*Corresponding author

Additional spectral data, detailed description of the experiments performed, ^{1}H NMR of the derivatives 2a–d and crystallographic data

Table of Contents

1. Additional graphical material...S2
2. Experimental...S3
3. ^{1}H NMR of derivatives **2a–d** and **6** S12
4. Crystallographic information S15

1. Additional graphical material

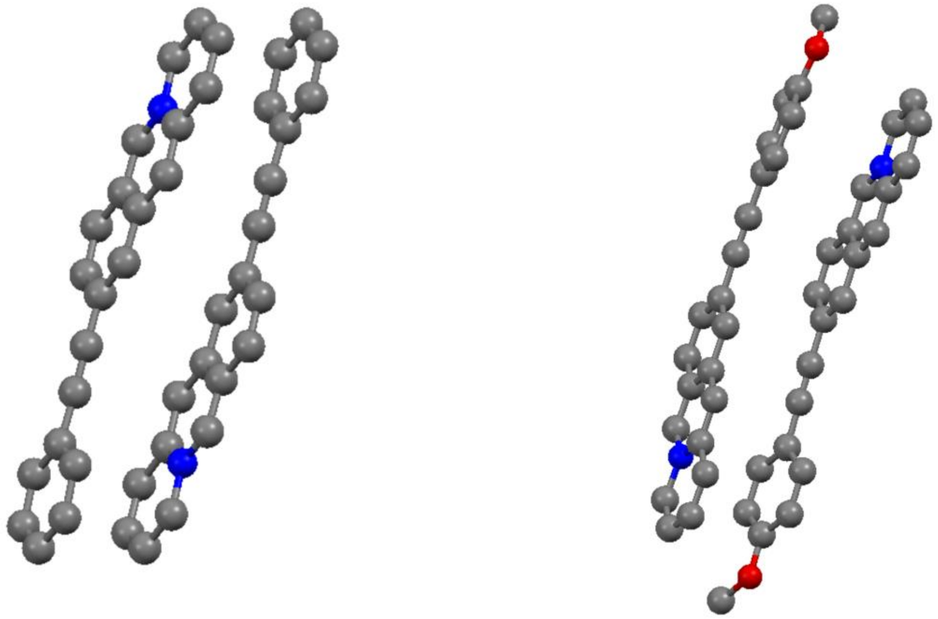

,

Figure S1: The structure of **2a** (left) and **2b** (right) in the solid state. Grey: C, blue: N, red: O; counter ions and H were removed for clarity.

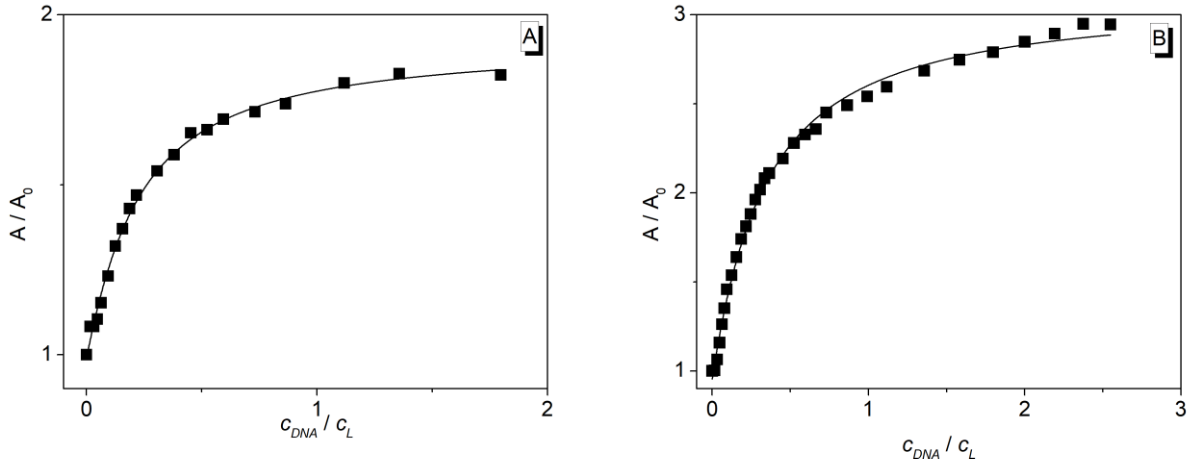

Figure S2: Plot of the relative absorbance, A/A_0 *versus* c_{DNA}/c_L from the titration of derivatives **2b** (A) and **2c** (B) with G4-DNA **22AG**. Lines denote the best fit of experimental data to the theoretical model.

2. Experimental

Materials

Oligodeoxyribonucleotide (HPLC purified) **22AG** [d($AG_3T_2AG_3T_2AG_3T_2AG_3$)] was purchased from Metabion Int. AG (Planegg/Martinsried). Calf thymus DNA (ct DNA) was purchased from Sigma-Aldrich (St. Louis, USA). The nucleic acids were used without further purification. Concentration of **22AG** is given as $c_{\text{oligonucleotide}}$; concentration of ct DNA is given in base pairs (bp). The G4-DNA **22AG** was dissolved in K-phosphate buffer, heated to 95 °C for 5 min and cooled slowly to room temperature in 4 h. K-phosphate buffer: 25 mM K_2HPO_4, 70 mM KCl; adjusted with 25 mM KH_2PO_4 to pH 7.0; BPE buffer: 6.0 mM Na_2HPO_4, 2.0 mM NaH_2PO_4, 1.0 mM Na_2EDTA; pH 7.0.

Equipment

Absorption spectroscopy: Varian Cary 100 Bio Spectrophotometer; emission spectroscopy: Varian Cary Eclipse; CD spectroscopy: Chirascan CD-Spectrometer (Aplied Photophysics); ^{1}H NMR spectroscopy: Bruker Avance 400 (^{1}H: 400 MHz, ^{13}C: 100 MHz). Spectra were analysed with software ACD/NMR Processor Academic Edition 12.02. The solvent residual signal of DMSO-d_6 was used as internal reference (^{1}H = 2.50, ^{13}C = 39.5; chemical shifts are given in ppm). Elemental analysis: HEKAtech EUROES combustion analyzer. Mass spectra (ESI) were recorded on a Finnigan LCQ Deca (U = 6 kV; working gas: argon; auxiliary gas: nitrogen; temperature of the capillary: 200 °C) and APCI-MS were recorded on Advion MS. Melting points (uncorrected): Büchi 545 (Büchi, Flawil, CH)

Methods

Spectroscopic titrations were conducted in thermostated quartz cuvettes at 20 °C. Titrant solutions were prepared directly prior to the experiment by dilution of the corresponding stock solution. Spectrometer slit width: 2 nm (photometry) and 5 nm (fluorimetry). Fluorescence spectra were smoothed by a factor of 5. The acid–base titrations and the determination of the pK_a value of the compound **2c** were performed according to published procedure [1]. The binding constants were determined by fitting the binding isotherms, Abs / Abs_0 *vs* c_{DNA} / c_L or I / I_0 *vs* c_{DNA} / c_L, from the fluorimetric or photometric titrations to the theoretical independent-site model (Equation 1) [2].

$$y = 1 + \frac{Q-1}{2}\left(A + nx + 1 - \sqrt{(Q + nx + 1)^2 - 4nx}\right) \quad (1)$$

Where $y = \frac{I}{I_0}$ or $\frac{Abs}{Abs_0}$;

$Q = \frac{I_\infty}{I_0}$ = the minimal fluorescence intensity in the presence of excess ligands;

$A = \frac{1}{K_b \times c_L}$;

$x = \frac{c_{DNA}}{c_L}$ = titration variable.

Synthesis

Synthesis of 9-aryl-substituted benzo[*b*]quinolizinium derivatives 1a–d

*Synthesis of benzo[b]quinolizinium-9-trifluroborate (**3b**)*

A solution of benzo[*b*]quinolizinium-9-boronic acid bromide (**3a**, 2.4 g, 7.8 mmol) [3] and $NaBF_4$ (1.5 g, 14 mmol) in methanol (45 mL) was stirred at room temperature for 6 h. The precipitate was filtered off and washed with methanol (2 × 10 mL) and diethyl ether (2 × 10 mL) to give the product as yellow solid (1.1 g, 4.5 mmol, 57%); mp = 254 °C

(dec.) – ^{1}H NMR (400 MHz, DMSO-d_6): δ = 7.82 (td, ^{3}J = 7 Hz, ^{4}J = 1 Hz, 1H, 3-H), 7.93 (td, ^{3}J = 8 Hz, ^{4}J = 1 Hz, 1H, 2-H), 8.04 (d, ^{3}J = 9 Hz, 1H, 7-H), 8.19–8.25 (m, 2H, 8-H, 10-H), 8.43 (d, ^{3}J = 9 Hz, 1H, 1-H), 9.05 (s, 1H, 11-H), 9.16 (d, ^{3}J = 7 Hz, 1H, 4-H), 10.26 (s, 1H, 6-H). – ^{13}C NMR (100 MHz, DMSO-d_6): δ = 121.2 (3-C), 123.3 (10-C), 125.2 (8-C), 125.4 (10a-C), 126.6 (1-C), 127.8 (11-C), 129.7 (2-C), 133.7 (4-C), 135.4 (11a-C), 136.1 (7-C), 136.9 (6a-C), 139.3 (6-C), 159.6 (9-C). – ^{19}F NMR (564 MHz, DMSO-d_6): δ = –140.8. – MS (ESI$^+$): *m/z* (%) = 247 (100) $[M]^+$

*Synthesis of benzenediazonium tetrafluoroborate (**4a**)*

The benzenediazonium tetrafluoroborate (**4a**) was prepared according to published procedure [4] from aniline (930 mg, 10 mmol) and $NaNO_2$ (696 mg, 10 mmol) to give the final product as white powder (1.6 g, 8.3 mmol, 83%) whose ^{1}H NMR spectroscopic data matched the reported ones [4]; mp = 87 °C (dec.).

*Synthesis of 4-methoxyphenyldiazonium tetrafluoroborate (4**b**)*

The 4-methoxyphenyldiazonium tetrafluoroborate (**4b**) was prepared according to published procedure [5] from aniline (1.2 g, 10 mmol) and *tert*-butyl nitrite (2.7 mL, 2.3 g, 23 mmol) to give the product as white powder (2.1 g, 9.4 mmol, 94%) whose ^{1}H-NMR spectroscopic data matched the reported ones [6].

*Synthesis of 4-(dimethylamine)phenyldiazonium tetrafluoroborate (**4c**)*

The 4-(dimethylamino)phenyldiazonium tetrafluoroborate (**4c**) was prepared according to published procedure [5] from aniline (1.2 g, 10 mmol) and *tert*-butyl nitrite (2.7 mL, 2.3 g, 23 mmol) to give the product as dark violet powder (2.3 g, 9.9 mmol, 99%) whose ^{1}H NMR spectroscopic data matched the reported ones [7]; mp = 144–145 °C.

*Synthesis of pyridine-4-diazonium tetrafluoroborate (**4d**)*

The pyridine-4-diazonium tetrafluoroborate (**4d**) was prepared according to published procedure [8] from 4-aminopyridine (4.0 g, 43 mmol) and $NaNO_2$ (3.1 g, 45 mmol) to give the product as white powder (7.8 g, 40 mmol, 95%) whose ^{1}H-NMR spectroscopic data matched the reported ones [8]; mp = 102 °C.

*General procedure I for the synthesis of 9-arylbenzo[b]quinolizinium derivatives **1a**–**d***

A mixture of the benzo[*b*]quinolizinium-9-trifluoroborate (**3b**, 1.0 equiv) and the diazonium salt (1.0 equiv) in water was freed from oxygen by three freeze-pump-thaw cycles and argon as inert gas. $[Pd(OAc)_2]_3$ (5.0 mol %) was added, and the solution was stirred under argon-gas atmosphere. The progress of the reaction mixture was monitored by thin-layer chromatography (SiO_2, $CHCl_3$/MeOH 9:1), and the diazonium salt (1.0 equiv) was further added in intervals of 24 h until all of the benzo[*b*]quinolizinium-9-trifluoroborate (**3b**) was consumed. The reaction mixture was filtered through celite, washed with acetonitrile (3 × 10 mL) and concentrated in vacuo to give the crude product, which is further purified by column chromatography or crystallization.

*9-Phenylbenzo[b]quinolizinium tetrafluoroborate (**1a**)*

According to the general procedure I, benzo[*b*]quinolizinium-9-trifluoroborate (**3b**, 250 mg, 1.0 mmol) and the diazonium salt (**4a**, 230 mg, 1.2 mmol) were treated according to general procedure I. The solvent was evaporated in vacuum and the crude product was purified with column chromatography (Silica, CH_2Cl_2/MeOH 9:1) to give the product as yellow crystals (149 mg, 0.43 mmol, 43%) whose ^{1}H NMR spectroscopic data match the reported ones [9]; mp = 204–205 °C (Ref. [9] 203–204 °C)

*9-(p-Methoxyphenyl)benzo[b]quinolizinium tetrafluoroborate (**1b**)*

Benzo[*b*]quinolizinium-9-trifluoroborate (**3b**, 250 mg, 1.0 mmol) and the diazonium salt (**4a**, 225 mg, 1.0 mmol) were treated according to general procedure I. The crude product was crystallized from acetonitrile to give the product as yellow crystals (365 mg, 0.95 mmol, 95%) whose ^{1}H NMR spectroscopic data matched the reported ones [9]; mp = 219–220 °C (Ref. [9] 218–220 °C).

*9-(p-N,N-Dimethylaminophenyl)benzo[b]quinolizinium tetrafluoroborate (**1c**)*

Benzo[*b*]quinolizinium-9-trifluoroborate (**3b**, 250 mg, 1.0 mmol) and the diazonium salt (**4c**, 235 mg, 1.0 mmol) were treated according to General Procedure I. The crude product was purified by column chromatography (Silica, $CHCl_3$: MeOH; 9:1) and further crystallized from acetonitrile to obtain the product as dark-red crystals (141 mg, 0.44 mmol, 44%) whose ^{1}H-NMR spectroscopic data matched the reported ones [9]; mp > 300 °C (Ref. [9] > 300 °C).

*9-(4-Pyridyl)benzo[b]quinolizinium tetrafluoroborate (**1d**)*

A solution of benzo[*b*]quinolizinium-9-trifluoroborate (**3b**, 250 mg, 1.0 mmol) and the diazonium salt (**4d**, 1.4 g, 7.0 mmol) in dry DMF (10 mL) was freed from oxygen by passing through a stream of argon-gas for 20 min. $Pd(PPh_3)_4$ (58 mg, 5.0 μmol) was added and the reaction mixture was heated at 80 °C for 7 days and the diazonium salt (1.0 equiv) was added in portions in intervals of 24 h. The reaction mixture was filtered through celite, washed with acetonitrile (3 × 10 mL) and the solvent was removed in vacuo to give the crude product that was washed with methanol and dried in vacuum to give the product as yellow solid (56 mg, 16 μmol, 16%). – ^{1}H NMR (400 MHz, DMSO-*d*$_6$): δ = 7.50–7.56 (m, 2H, 3’-H, 5’-H), 7.71 (d, ^{3}J = 4 Hz, 2H, 2’-H, 6’-H), 7.95–8.01 (m,

1H, 3-H), 8.02–8.07 (m, 1H, 2-H), 8.08–8.15 (m, 1H, 8-H), 8.49–8.52 (m, 1H, 7-H), 8.58–8.62 (m, 1H, 1-H), 8.63 (s, 1H, 10-H), 9.18 (s, 1H, 11-H), 9.25–9.31 (m, 1H, 4-H), 10.40 (s, 1H, 6-H).

Synthesis of 9-(arylethynyl)benzo[*b*]quinolizinium derivatives 2a–d

*Synthesis of (phenylethynyl)copper (**6**)*

The (phenylethynyl)copper (**6**) was prepared according to published procedure [10] from phenylacetylene (1.1 mL, 10 mmol) and $CuSO_4(H_2O)_5$ (2.5 g, 10 mmol) to give the product as yellow solid (162 mg, 98%); mp = 238–239 °C. (Ref. [11] 239–240 °C) – ^{1}H NMR (400 MHz, DMSO-d_6): δ = 7.45 (d, ^3J = 7 Hz, 1H, 3-H, 5-H) 7.49 (d, ^3J = 7 Hz, 1H, 4-H), 7.62 (dd, ^3J = 7 Hz, ,^4J = 2 Hz, 1H, 2-H, 6-H).

This compound is known [10,11], however, the ^{1}H NMR data are not reported.

*Synthesis of 9-(phenylethynyl)benzo[b]quinolizinium hexafluorophosphate (**2a**)*

A mixture of 9-iodobenzo[b]quinolizinium bromide [12] (**5**, 250 mg, 0.65 mmol) and 9-(phenylethynl)copper (**6**, 118 mg, 0.72 mmol) in anhydrous DMF (10 mL) was freed from oxygen by three freeze-pump-thaw cycles and argon as inert gas. Tetrakis(triphenylphospine)palladium (37 mg, 32 μmol) was added and the reaction mixture was stirred for 18 h at room temperature. Water (30 mL) was added to the reaction mixture, which was filtered and washed with methanol (30 mL). To the filtrate, a saturated solution of NH_4PF_6 (3 mL) was added and the yellow precipitate was filtered and washed with cold water (10 mL). The crude product was purified by crystallization from acetone to provide the pure product as yellow needles (130 mg, 0.30 mmol, 47%); mp = 260–261 °C. – ^{1}H NMR (400 MHz, DMSO-d_6): δ = 7.52–7.54 (m, 3H, 3'-H, 4'-H, 5'-

H), 7.69–7.72 (m, 2H, 2’-H, 6’-H), 7.96–7.99 (m, 1H, 3-H), 8.04 (dd, ^{3}J = 9 Hz, ^{4}J = 1 Hz, 1H, 8-H), 8.10 (m, 1H, 2-H), 8.51 (d, ^{3}J = 9 Hz, 1H, 7-H), 8.61 (d, ^{3}J = 9 Hz, 1H, 1-H), 8.63 (s, 1H, 10-H), 9.18 (s, 1H, 11-H), 9.28 (d, ^{3}J = 7 Hz, 1H, 4-H), 10.41 (s, 1H, 6-H). – ^{13}C NMR (100 MHz, DMSO-d_6): δ (ppm) = 88.9 (2’’-C), 96.0 (1’’-C), 121.5 (1’-C), 123.0 (3-C), 124.8 (11-C), 125.2 (6a-C), 127.4 (1-C), 128.3 (9-C), 129.0 (7-C), 129.4 (3’-C), 130.2 (10-C), 130.4 (4’-C, 5’-C), 132.1 (2-C), 132.3 (2’-C, 6’-C), 132.8 (8-C), 135 (4-C), 135.3 (10a-C), 138.4 (11a-C), 140.4 (6-C). – MS (ESI$^+$): *m*/*z* (%) = 280 (100) [M–PF_6]$^+$. – El. Anal. for $C_{21}H_{14}F_6NP$ (425.08), calcd (%): C 59.30, H 3.32, N 3.29, found (%): C 59.40, H 3.59, N 3.01.

*General procedure II for the synthesis of benzo[b]quinolizinium derivatives **2b**–**d***

A mixture of 9-iodobenzo[*b*]quinolizinium bromide [12] (**6**, 1.0 equiv), bis(triphenylphosphine)palladium(II) dichloride (5 mol %), CuI (10 mol %) and triethylamine (1.0 equiv) in anhydrous DMF (7 mL) was stirred under nitrogen-gas atmosphere for 15 min at room temperature, followed by the addition of the corresponding acetylene derivative (1.1 equiv). The solution was stirred for 16 h at room temperature. The reaction mixture was concentrated under vacuum and the precipitate was filtered and dried under vacuum. The crude product was crystallized from methanol or CH_2Cl_2/acetone mixture to give the product.

*9-((4-Methoxyphenyl)ethynyl)benzo[b]quinolizinium bromide (**2b**)*

According to general procedure II obtained from 9-iodobenzo[*b*]quinolizinium bromide [12] (**6**, 200 mg, 0.77 mmol) and 1-ethynyl-4-methoxybenzene (**4b**, 113 mg, 0.85 mmol) as yellow needles (126 mg, 0.32 mmol, 42%); mp = 255–256 °C. – ^{1}H NMR (400 MHz,

DMSO-d_6): δ = 3.84 (s, 3H, CH_3), 7.07 (d, 2H, ^3J = 9 Hz, 2'-H, 6'H), 7.64 (d, ^3J = 9 Hz, 2H, 3'-H, 5'H), 7.95–8.02 (m, 2H, 3-H, 8-H), 8.08–8.12 (m, 1H, 2-H), 8.48 (d, ^3J = 9 Hz, 1H, 7-H), 8.56 (s, 1H, 10-H), 8.59 (d, ^3J = 9 Hz, 1H, 1-H), 9.16 (s, 1H, 11-H), 9.28 (d, ^3J = 7 Hz, 1H, 4-H), 10.43 (s, 1H, 6-H). – ^{13}C NMR (100 MHz, DMSO-d_6): δ = 55.4 (CH_3), 89.9 (2''-C), 87.7 (1''-C), 112.9 (2'-C, 4'-C), 114.7 (5'-C), 122.5 (10-C), 124.1 (11-C), 124.6 (3-C), 126.9 (8-C, 9-C), 128.5 (6a-C), 129.1 (7-C), 131.6 (1'-C, 5'-C), 132.5 (1-C), 133.7 (2-C), 134.5 (11a-C), 134.9 (4-C), 137.9 (10a-C), 139.9 (6-C), 161.5 (3'C). – APCI-MS: *m/z* (%) = 388 [M-H]. – El. Anal. for $C_{22}H_{16}BrNO$ (389.04), calcd. (%): C 67.71, H 4.13, N 3.59, found (%): C 67.52, H 4.09, N 3.65.

*9-((4-(Dimethylamino)phenyl)ethynyl)benzo[b]quinolizinium bromide (**2c**)*

According to general procedure II obtained from 9-iodobenzo[*b*]quinolizinium bromide [12] (**6**, 200 mg, 0.77 mmol) and 1-ethynyl-*N*,*N*-dimethylaniline (**4c**, 123 mg, 0.85 mmol) as red needles (181 mg, 0.54 mmol, 70%); mp = 272–273 °C. ^{1}H NMR (400 MHz, DMSO-d_6): δ = 3.00 (s, 6H, 2 x CH_3), 6.76 (d, ^3J = 9 Hz, 2H, 3'-H, 5'-H), 7.47 (d, ^3J = 9 Hz, 2H, 2'-H, 6'-H), 7.91–7.97 (m, 2H, 3-H, 8-H), 8.05–8.09 (m, 1H, 2-H), 8.42–8.45 (m, 2H, 7-H, 10-H), 8.54 (d, ^3J = 9 Hz, 1H, 1-H), 9.08 (s, 1H, 11-H), 9.23 (d, ^3J = 7 Hz, 1H, 4-H), 10.33 (s, 1H, 6-H). – ^{13}C NMR (75 MHz, DMSO-d_6) : δ = 40.4 (2 x CH_3), 88.2 (2''-C), 99.3 (1''-C), 106.9 (6'-H),112.1 (2'-C, 4'-C), 122.5 (10-C), 123.9 (11-C), 124.7 (3-C), 127.9 (1-C), 128.1 (8-C), 128.6 (6a-C), 129.5 (7-C), 131.7 (1'-C), 132.8 (5'-C), 133.5 (9-C), 134.7 (10a-C), 135.4 (2-C), 138.2 (4-C), 139.9 (6-C) 151.1 (3'-C). – APCI-MS: *m/z* = 279 $[M-N(CH_3)_2]^+$, 290 $[M-CH_3]^+$. – El. Anal. for $C_{23}H_{19}BrN_2$ (402.07), calcd. (%): C 68.74, H 5.53, N 6.68, found (%): C 68.54, H 5.35, N 6.82.

*9-(Phenanthrene-9-ylethynyl)benzo[b]quinolizinium bromide (**2d**)*

According to general procedure II obtained from 9-iodobenzo[*b*]quinolizinium bromide [12] (**6**, 200 mg, 0.77 mmol) and 9-ethynylphenanthrene (**4d**, 172 mg, 0.85 mmol) as yellow crystals (216 mg, 0.47 mmol, 62%; mp = 246–248 °C). – ^{1}H NMR (400 MHz, DMSO-d_6): δ = 7.70–7.80 (m, 2H, 6'-H, 7'-H), 7.82–7.88 (m, 2H, 1'-H, 8'-H), 7.96–8.00 (m, 1H, 3-H), 8.07–8.12 (m, 2H, 2-H, 8-H), 8.19 (dd, ^{3}J = 9, ^{4}J = 2 Hz, 1H, 5'-H), 8.37 (s, 1H, 10-H), 8.49 (d, ^{3}J = 9, 1H, 1-H), 8.52–8.58 (m, 2H, 2'-H, 3'-H), 8.74 (s, 1H, 10'-H), 8.84 (d, ^{3}J = 8, 1H, 7-H), 8.91 (dd, ^{3}J = 8, ^{4}J = 2 Hz, 1H, 4'-H), 9.14 (s, 1H, 11-H), 9.26 (d, ^{3}J = 7 Hz, 1H, 4-H), 10.37 (s, 1H, 6-H). – ^{13}C NMR (100 MHz, DMSO-d_6): δ (ppm) = 93.2 (2''-C), 93.6 (1''-C), 117.3 (1'-C), 122.6 (2'-C), 122.9 (10-C, 11-C), 123.5 (7'-C), 124.3 (3-C), 124.7 (1-C), 126.2 (9-C), 126.9 (8'-C), 127.5 (6a-C), 127.8 (7-C) 128.6 (8-C), 128.7 (6'-C), 128.9 (5'-C), 129.5 (4'-C), 129.8 (3'-C), 129.9 (6a'-C) 130.1 (6b'-C), 130.4 (10a'-C), 131.6 (2a'-C), 132.4 (9'-C) 133.3 (11'-C), 134.5 (10a-C), 134.8 (2-C), 137.8 (4-C), 139.7 (6-C). – APCI-MS: *m*/*z* = 474 [M]. – El. Anal. for $C_{30}H_{22}BrN$ (459.06), calcd. (%): C 75.63, H 4.65, N 2.94, found (%) C 75.21, H 4.12, N 3.01.

3. ^{1}H NMR of derivatives 2a–d and 6

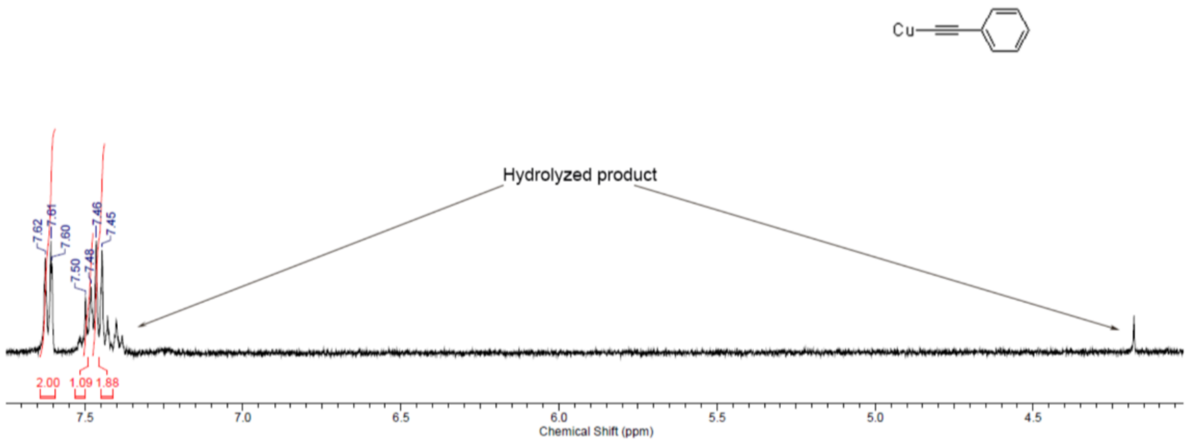

Figure S3: ^{1}H NMR spectrum (400 MHz) of (phenylethynyl)coper (**6**) in DMSO-d_6.

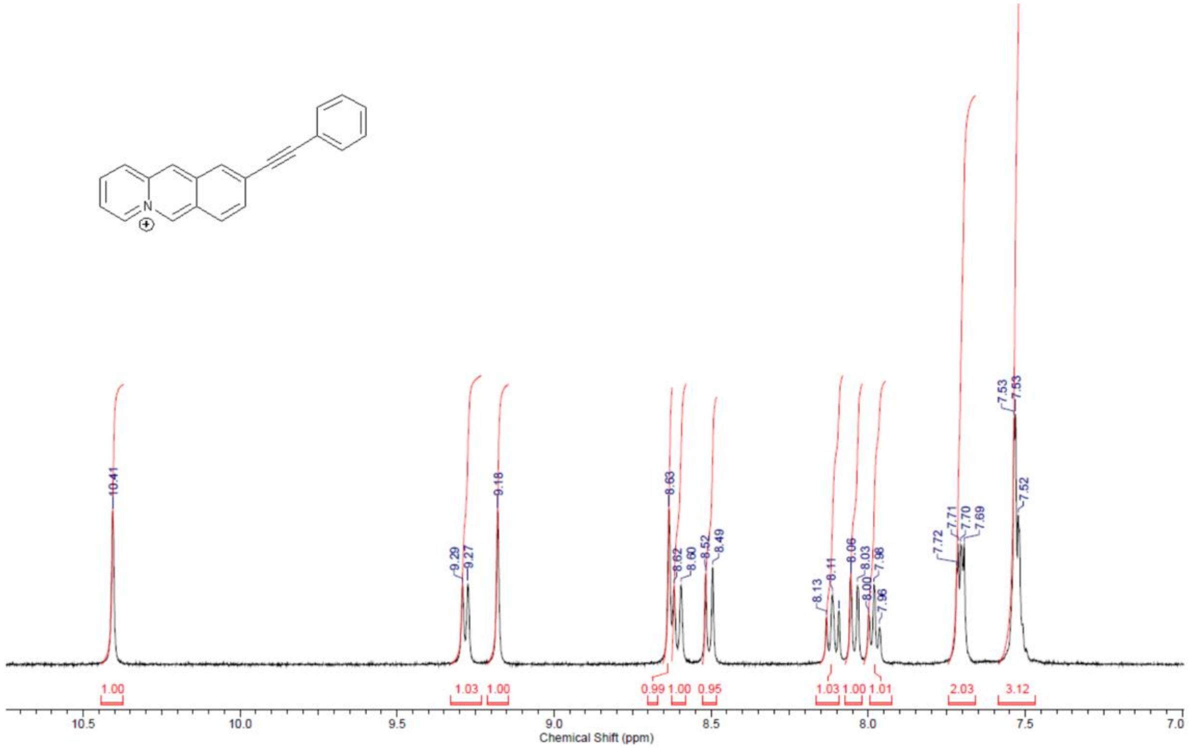

Figure S4: ^{1}H NMR spectrum (400 MHz) of 9-(phenylethynyl)benzo[*b*]quinolizinium (**2a**) in DMSO-d_6.

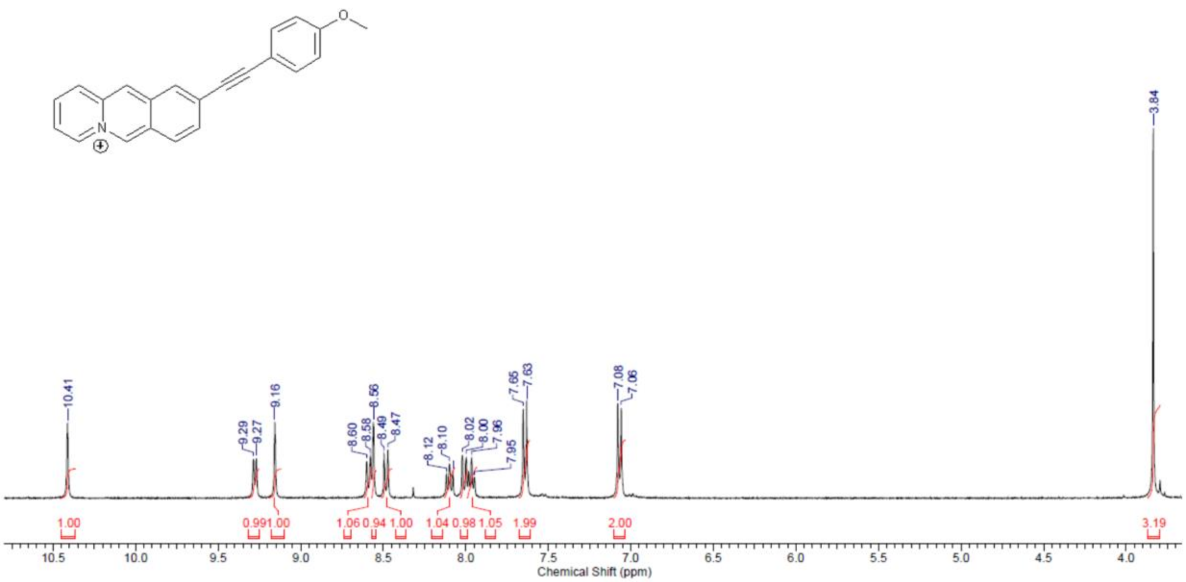

Figure S5: ^{1}H NMR spectrum (400 MHz) of 9-((4-meothoxyphenyl)ethynyl)benzo[*b*]quinolizinium (**2b**) in DMSO-d_6.

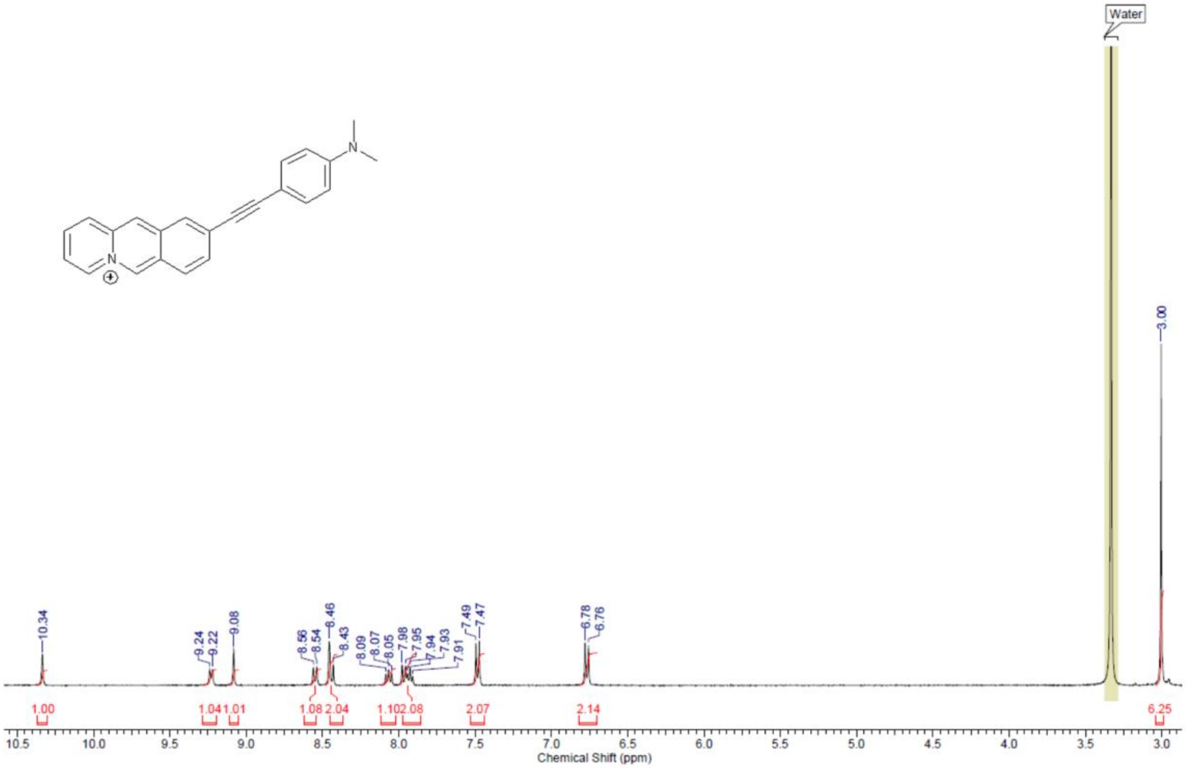

Figure S6: ^{1}H NMR spectrum (400 MHz) of 9-((4-(dimethylamion)phenyl)ethynyl) benzo[*b*]quinolizinium (**2c**) in DMSO-d_6.

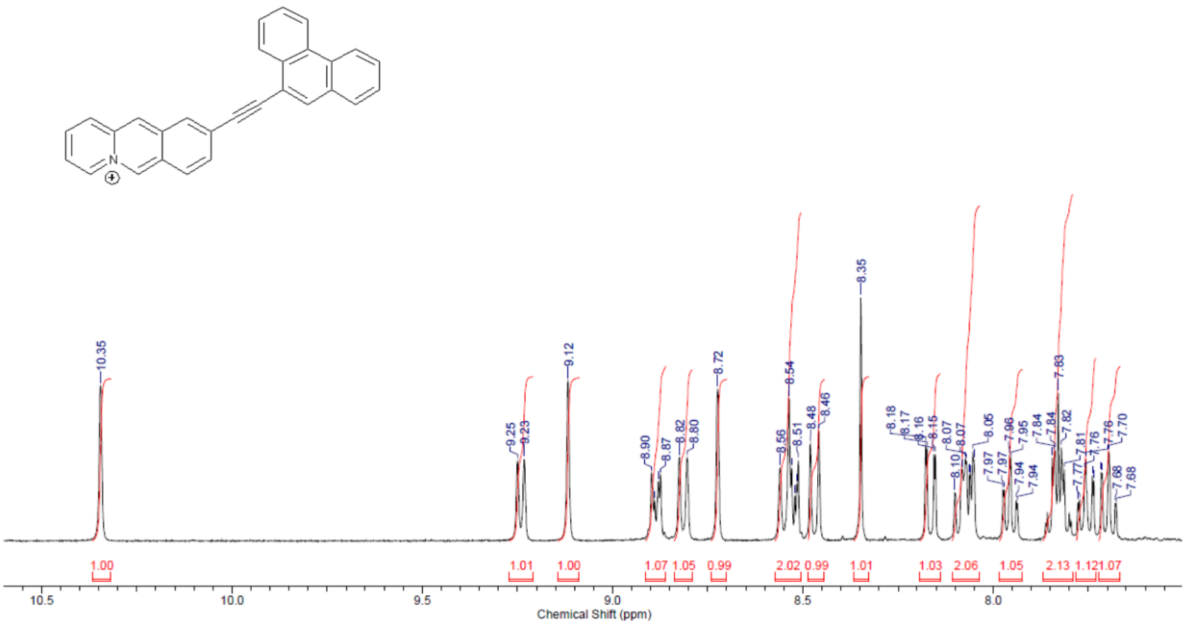

Figure S7: ^{1}H NMR spectrum (400 MHz) of 9-(phenanthrene-9-ylethynyl)benzo[*b*]quinolizinium (**2d**) in DMSO-d_6.

4. Crystallographic Information

Diffraction data for **2a** and **2b** were collected at low temperature (−103.0 °C) with a STOE-IPDS 2T diffractometer with graphite-monochromated molybdenum Kα radiation, λ = 0.71073 Å. The structures were solved by direct methods (SHELXT [13]) and refined by full matrix least-squares method based on F^2 (WINGX GUI [14] and SHELXL [15] programs). The data quality for **2a** was compromised due to twinning plus disorder of the hexafluorophosphate anion (rotation of four equatorial fluorines around a F-P-F axis). The disorder was treated using rather hard constraints (SADI, SIMU, DELU). The twinning was handled by generating and using a hklf5 file for the refinement. In addition, approximately one lattice solvent dmf was so severely disordered that no atom positions could be assigned. The fraction of solvent was treated as diffuse contribution to the overall scattering and was removed with the SQUEEZE/PLATON procedure [16]. Furthermore, the absorption corrected hkl file was cut at 1.0 Å resolution as beyond that (<1.0 Å) the data to noise ratio was too poor to be considered. All this gives rise to A and B alerts in the check cif file. In **2b** the lattice solvent has an occupancy of only 80%; i.e., not all potential positions in the cells are actually occupied. The occupancy was derived using a free variable and then fixed to the average value found. All non-hydrogen-atoms were refined with anisotropic displacement parameters. All hydrogen atoms were refined isotropically on calculated positions using a riding model with their U_{iso} values constrained to 1.5 U_{eq} of their pivot atoms for terminal sp^3 carbon atoms and 1.2 times for the aromatic carbon atoms. All crystallographic data were deposited with the Cambridge Crystallographic Data Centre, CCDC, and can be obtained free of charge on quoting the depository numbers CCDC 1837474 (**2a**) and 1837475 (**2b**) by

FAX (+44-1223-336-033), email (deposit@ccdc.cam.ac.uk) or their web interface (at http://www.ccdc.cam.ac.uk).

Table S1: Crystal data and structure refinement details of **2a** and **2b**

	**2a**	**2b**
Molecular Formula	$C_{21}H_{14}NF_6P$	$C_{22.80}H_{16.8}BrCl_{2.4}NO$
Temperature	170(2) K	170(2) K
Crystal Size	0.467 x 0.126 x 0.071 mm	0.233 x 0.057 x 0.035 mm
Crystal System	Triclinic	Monoclinic
Lattice Parameters	*a* = 7.0107(14) Å	*a* = 13.979(3) Å
	b = 10.444(2) Å	*b* = 10.354(2) Å
	c = 14.838(3) Å	*c* = 30.522(6) Å
	α = 99.42(3)°	α = 90°
	β = 97.29(3)°	β = 96.59(3)°
	γ = 109.69(3)°	γ = 90°
Volume	1014.9(3) $Å^3$	4388.6(16) $Å^3$
Space Group	$P\bar{1}$	*I* 2/a
Z value	2	8
Calculated density	1.392 mg / m^3	1.470 mg / m^3
Absorption coefficient	0.195 mm^{-1}	2.179 mm^{-1}
F(000)	432	1955
Refinement	Full-matrix least-squares on F^2	Full-matrix least-squares on F^2
Residuals (refined on F^2, all data): *R*1; *wR*2	0.1587; 0.3932	0.0614, 0.1529
Maximum peak in Final Diff. Map	0.440 $e^-/Å^3$	1.160 $e^-/Å^3$
Minimum peak in Final Diff. Map	-0.477 $e^-/Å^3$	–0.533 $e^-/Å^3$

References

1. Granzhan, A.; Ihmels, H.; Viola, G. *J. Am. Chem. Soc.* **2007**, *129*, 1254–1267.

2. Stootman, F. H.; Fisher, D. M.; Rodger, A.; Aldrich-Wright, J. R. *Analyst* **2006**, *131*, 1145–1151.

3. Tian, M.; Ihmels, H. *Synthesis* **2009**, 4226–4234.

4. Lackner, A. D.; Fürstner, A. *Angew. Chem. Int. Ed.* **2015**, *54*, 12814–12818.

5. Wu, J.; Gu, Y.; Leng, X.; Shen, Q. *Angew. Chem. Int. Ed.* **2015**, *54*, 7648–7652

6. Bonin, H.; Delbrayelle, D.; Demonchaux, P.; Gras, E. *Chem. Commun.* **2010**, *46*, 2677–2679

7. Nikolaienko, P.; Rueping, M. *Chem. Eur. J.* **2016**, *22*, 2620–2623

8. Naidek, K. P.; Hoffmeister, D. M.; Pazinato, J.; Westphal, E.; Gallardo, H.; Nakamura, M.; Araki, K.; Toma, H. E.; Winnischofer, H. *Eur. J. Inorg. Chem.* **2014**, *2014*, 1150–1157.

9. Tian, M.; Ihmels, H. *Synthesis* **2009**, 4226–4234.

10. Gallego, D.; Brück, A.; Irran, E.; Meier, F.; Kaupp, M.; Driess, M.; Hartwig, J. F. *J. Am. Chem. Soc.* **2013**, *135*, 15617–15626.

11. Shao, C.; Cheng, G.; Su, D.; Xu, Jimin; Wang, X.; Hu, Y. *Adv. Synth. Catal.* **2010**, *352*, 1587–1592

12. Bradsher, C. K.; Sherer, J. P.; Parham, J. H. *J. Chem. Eng. Data* **1965**, *10*, 180–183.

13. Sheldrick, G. *Acta Cryst. A* **2015**, *71*, 3–8.

14. Farrugia, L. *J. Appl. Crystallogr.* **2012**, *45*, 849–854.

15. Sheldrick, G. *Acta Cryst. C* **2015**, *71*, 3–8.

16. Spek, A. L. *Inorg. Chim. Acta* **2018**, *470*, 232–237
